# Characterisation and Expression of Calpain Family Members in Relation to Nutritional Status, Diet Composition and Flesh Texture in Gilthead Sea Bream (*Sparus aurata*)

**DOI:** 10.1371/journal.pone.0075349

**Published:** 2013-09-25

**Authors:** Cristina Salmerón, Daniel García de la serrana, Vanesa Jiménez-Amilburu, Ramón Fontanillas, Isabel Navarro, Ian A. Johnston, Joaquim Gutiérrez, Encarnación Capilla

**Affiliations:** 1 Department of Physiology and Immunology, Faculty of Biology, University of Barcelona, Barcelona, Spain; 2 Scottish Oceans Institute, School of Biology, University of St Andrews, St Andrews Fife, Scotland, United Kingdom; 3 Skretting Aquaculture Research Centre, Stavanger, Norway; Emory University, United States of America

## Abstract

Calpains are non-lysosomal calcium-activated neutral proteases involved in a wide range of cellular processes including muscle proteolysis linked to *post-mortem* flesh softening. The aims of this study were (a) to characterise several members of the calpain system in gilthead sea bream and (b) to examine their expression in relation to nutritional status and muscle tenderisation. We identified the complete open reading frame of gilthead sea bream calpains1-3, *sacapn1*, *sacapn2*, *sacapn3*, and two paralogs of the calpain small subunit1, *sacapns1a* and *sacapns1b*. Proteins showed 63–90% sequence identity compared with sequences from mammals and other teleost fishes, and the characteristic domain structure of vertebrate calpains. Transcripts of *sacapn1*, *sacapn2*, *sacapns1a* and *sacapns1b* had a wide tissue distribution, whereas *sacapn3* was almost exclusively detected in skeletal muscle. Next, we assessed transcript expression in skeletal muscle following alteration of nutritional status by (a) fasting and re-feeding or (b) feeding four experimental diets with different carbohydrate-to-protein ratios. Fasting significantly reduced plasma glucose and increased free fatty acids and triglycerides, together with a significant increase in *sacapns1b* expression. Following 7 days of re-feeding, plasma parameters returned to fed values and *sacapn1*, *sacapn2*, *sacapns1a* and *sacapns1b* expression was significantly reduced. Furthermore, an increase in dietary carbohydrate content (11 to 39%) diminished growth but increased muscle texture, which showed a significant correlation with decreased *sacapn1* and *sacapns1a* expression, whilst the other calpains remained unaffected. This study has demonstrated that calpain expression is modulated by nutritional status and diet composition in gilthead sea bream, and that the expression of several calpain members is correlated with muscle texture, indicating their potential use as molecular markers for flesh quality in aquaculture production.

## Introduction

Gilthead sea bream (*Sparus aurata*) is an important marine species reared in the Mediterranean area. In 2011, 94,4% of its production came from farms (151.346Tn), whereas only 5,6% (8.330Tn) came from extractive fishing [1]. The axial musculature or fillet is the main product of aquaculture and in gilthead sea bream represents approximately 65% of body mass.

While in terrestrial farmed animals meat tenderisation is a desirable process, in farmed fish freshness and firm texture are considered among the most important quality attributes of the flesh. Texture is influenced by various physical, chemical, biochemical and microbiological changes that may occur *post-mortem*, finally resulting in a loss of quality. An important determinant of flesh texture is muscle cellularity i.e. the number and size distribution of the fibres [2], [3]. These parameters can be affected by a number of factors such as diet [4], [5], [6], photoperiod [7], temperature [8], [9], [10] and exercise training [11]–[14].

Muscle texture also depends on the ratio between protein synthesis and degradation. During the last decades, the activity of muscle endogenous proteinases has received a great deal of attention due to their role in muscle protein turnover and growth, and *post-mortem* proteolysis. The major intracellular proteolytic systems include the proteasome, calpains, cathepsins and caspases. Currently, calpains and cathepsins (lysosomal proteases) are known to hydrolyse myofibrillar proteins, and all available evidence indicate that the structural changes that take place during *post-mortem* storage of meat are caused by muscle proteases, especially members of these two families [15].

Calpains are Ca^2+^-dependent intracellular proteases that belong to the papain superfamily of cysteine proteases and are found in almost all eukaryotes and a few bacteria, but not in archaebacteria [16]. The human genome contains 15 genes that encode calpains. Nine of them encode the classical calpains, Calpain1 to Calpain3, Calpain8, Calpain9, and Calpain11 to Calpain14. All of them contain a C2-like (CL2) and a penta EF-hand (PEF) domain plus the calpain-like protease (CysPc) domain. The remaining non-classical calpains (Calpain5 to Calpain7, Calpain10, Calpain15 and Calpain16) lack both, the CL2 and PEF domains [16]. Depending on their expression across tissues, classical calpains are classified in humans as ubiquitous (*calpain1*, *calpain2*, *calpain13* and *calpain14*) or tissue-specific (*calpain3* in skeletal muscle, *calpain8* and *calpain9* in gastrointestinal tract, *calpain11* in testis and *calpain12* in hair follicle).

Defects in ubiquitous calpains may be lethal, as seen in *calpain2*–/– mice (*Mus musculus*) [17], whereas defects in tissue-specific calpains may cause tissue-specific phenotypes such as the muscular dystrophy caused by mutations in *calpain3* [18]. Calpain1 and Calpain2 have been widely studied in vertebrates; both are 80 kDa catalytic subunits that independently bind a common 30 kDa regulatory subunit (Calpain4 or calpain small subunit) to respectively form a heterodimer, which is named µ-calpain or m-calpain for its activation by micro or millimolar concentrations of Ca^2+^, respectively. A large number of proteins including cytoskeletal proteins, kinases, phosphatases, membrane-associated proteins, such as receptors or ion channels, and some transcription factors have been reported to be cleaved by calpains in *in vitro* assays [19]. Nevertheless, experimental evidence has clearly suggested that µ-calpain, but not m-calpain, has the most significant role in *post-mortem* proteolysis and meat tenderisation [20].

Previous studies in rainbow trout (*Oncorhynchus mykiss*) [21], Atlantic halibut (*Hippoglossus hippoglossus*) [22], zebrafish (*Danio rerio*) [23] and more recently in channel catfish (*Ictalurus punctatus*) [24] have shown that fish calpains have high amino acid identity and the characteristic domains of their orthologs in mammals. Retained paralogs of several calpain family members have been identified from the Whole Genome Duplication that occurred early in the adaptive radiation of the bony fishes [22], [25]. Teleosts also contain a ubiquitously expressed µ/m calpain which has one-to-one orthology with the testis-restricted *calpain11* in placental mammals [25]. It has also been reported that teleost calpains may be differentially regulated according to nutritional status. For example, in Atlantic halibut with 60 days of feed restriction, it was shown that *calpain1* transcript levels were significantly decreased after 7 days of re-feeding; at the same time that *calpain3* and *calpain11* expression significantly increased, whereas *calpain2-like* showed little response [22]. In channel catfish, 35 days of fasting increased the expression of *calpain2*, while decreasing that of *calpain1* and *calpain3* [24]. In another study in rainbow trout, fasting also for 35 days stimulated the expression of *calpain1*, *calpain2* and *calpastatin* (the endogenous specific inhibitor of ubiquitous calpains), suggesting a potential role for calpains in protein mobilization as a source of energy under catabolic conditions [21]. The same authors also observed that rainbow trout strains with reduced growth rate and softest fillet had significantly lower levels of *calpastatin* expression, but this softness effect related to the strain disappeared when fish were fed a high energy diet, indicating that diet also modulates calpain expression and texture [26]. Another study in sea bass (*Dicentrarchus labrax*) fed diets with three different levels of fat reported that a high lipid content in muscle could be responsible for faster *post-mortem* proteolysis, and suggested a possible activation of calpains related to lipid accumulation [27]. Further research is needed to elucidate the potential importance of diet, calpain expression and/or activity on texture in key aquaculture species.

The first objective of the present study was to identify and characterise different members of the calpain proteolytic system in gilthead sea bream. Secondly, in order to better understand the physiological situations that may regulate calpains expression in this species, transcript abundance was studied in fast-twitch skeletal muscle in response to: fasting/re-feeding conditions and various experimental diets with different ratios of protein and carbohydrate. Finally, the relationship between calpains expression and fillet firmness was also examined to determine their potential use as molecular markers of flesh quality.

## Materials and Methods

### Ethics statement

All animal handling procedures were approved by the Ethics and Animal Care Committee of the University of Barcelona (CEEA 239/09) and the Departament de Medi Ambient i Habitatge (DMAH permit number 5420, Generalitat de Catalunya, Spain) following the European Union, Spanish and Catalan Government-established norms and procedures.

### Animals and experimental trials

For the tissue screening experiment 10 juvenile gilthead sea bream (67.14±9.89 g, 15.86±0.88 cm fork length) from Tinamenor S.L (Pesués, Spain) were maintained at the facilities of the University of Barcelona (Barcelona, Spain), fed *ad libitum* twice a day with commercial pellets (Excel, Skretting, Burgos, Spain) and held at 21±1°C, pH of 7.5–8 in a recirculating seawater tank (400 L) with 12 h light:12 h dark photoperiod.

For the fasting/re-feeding experiment 120 juvenile gilthead sea bream (49.52±5.91 g) from the Institut de Recerca i Tecnologia Agroalimentàries (IRTA, Sant Carles de la Ràpita, Spain) were maintained at the facilities of the University of Barcelona (Barcelona, Spain) homogenously distributed in 8 recirculating seawater tanks (200 L) and held at 21±1°C, pH 7.5–8 with 12L:12D photoperiod. Fish were acclimated for a month and fed at 3% body weight twice a day (the ration was given 70% in the morning and 30% in the afternoon) with commercial pellets (Excel, Skretting, Burgos, Spain). First, 1 fish from each tank was sampled for time 0 (D0C). Then, fish were divided into two conditions: Control fed group (C) and Fasted group (F). The F group was fasted during 30 days while the C group was fed at 3% body weight for the duration of the whole experiment. Samples of 8 fish from each condition were collected at days 15 and 30 (D15C/F and D30C/F). Then, for the re-feeding experiment, fasted animals for 30 days (D0F) were re-fed at 2% body weight (lower than the control normal ration to facilitate correct adaptation of the digestive system) during 7 and 14 days and sampled (8 fish per condition at each time, D7R and D14R).

Finally, for the dietary experiment, 204 adult gilthead sea bream with an initial average weight of 115 g were maintained at IRTA facilities (Sant Carles de la Ràpita, Spain) and held at 22–24°C and natural photoperiod. Animals were homogenously distributed in 12 seawater tanks (17 fish/tank and 3 tanks per condition) connected to a closed recirculation system with feed collectors to measure the food wasted to calculate feed intake. Fish were fed *ad libitum* twice a day for 107 days, using automatic fish feeders, with four experimental diets containing different percentages in protein/carbohydrate (CH) (46/11, 46/19, 42/35 and 40/39, respectively) and 17% lipid (Table 1). At the end of the experiment, 9 fish of each group (3 fish per tank) were sampled for plasma constituents, biometrics, colour, texture and expression analysis. Together with the sampled fish, the remaining fish were also weighted to obtain the specific growth rate (SGR) and the feed conversion rate (FCR) values of all fish.

**Table 1:** Ingredients and chemical composition of the experimental diets.

Diet	46/11	46/19	42/35	40/39
**Raw materials (%)**				
Cellulose	17,07	7,70	0,00	0,00
Fish meal	20,00	20,00	20,00	20,00
Corn gluten	13,10	13,10	14,71	9,77
Wheat gluten	20,00	20,00	20,00	21,02
Fish oil	11,27	11,27	11,29	11,52
Soya concentrate	11,53	11,53	2,67	2,86
Mineral vitamin	2,00	2,00	2,00	2,00
Yttrium premix	0,10	0,10	0,10	0,10
Wheat Starch	5,00	14,18	29,00	33,00
Total	100	100	100	100
**Calculated (% dry matter)**				
Protein	46,85	47,32	43,33	41,12
Fat	17,03	17,21	17,33	17,31
Starch	7,56	16,31	30,29	36,22
**Analysed (% dry matter)**				
Protein	45,73	46,17	42,14	39,57
Fat	16,44	17,34	17,03	17,19
Starch	10,85	18,51	35,15	38,70

Skretting designed the diets and performed the nutrient analysis. Nomenclature of the diets corresponds to the percentage of protein/carbohydrate analysed.

Before sampling, all animals were fasted 24 h to avoid regurgitation of food and to obtain basal values of plasma metabolites and also to closely mimic the market situation, since this is a common practice before sacrificing commercial products for aquaculture. The fish were then anesthetised with tricaine methane sulphonate (MS-222 0.1 g/L, Sigma, Tres Cantos, Spain) and sacrificed with a blow on the head and medullar section. Blood from all fish of the dietary and fasting/re-feeding experiments was taken (1 mL/fish) from the caudal vein using 23G syringes with EDTA-Na and quickly centrifuged at 5000 rpm for 10 min to separate the plasma. Biometrics including body weight, total length, hepatosomatic index (HSI), mesenteric fat index (MFI) and condition factor (CF) were measured. Flesh colour was analysed and samples of fast skeletal muscle were either taken for texture measurement and kept on ice, or immediately snap-frozen in liquid nitrogen and stored at –80°C for gene expression analyses. The same procedure was used for 15 distinct tissue-types (fast muscle, slow muscle, fat, bone, head kidney, spleen, eye, brain, stomach, proximal intestine, distal intestine, pyloric caeca, skin, liver and heart) from 10 fish.

### Plasma parameters

Plasma glucose concentration was determined by a glucose oxidase colorimetric method (Spinreact, Sant Esteve d’en Bas, Spain). Plasma free fatty acids (FFAs) concentration was measured using a commercially available kit (NEFA-HR2, Wako Chemicals GmbH, Neuss, Germany). Plasma triglycerides (TGs) were hydrolysed by a lipase, and the released glycerol was measured by a peroxidase-coupled colorimetric assay (Spinreact, Sant Esteve d’en Bas, Spain).

### Flesh colour and texture

Muscle colour was measured at the time of sampling using a portable CR400 Chroma Meter (Konica Minolta, Madrid, Spain). The colorimeter was calibrated using the white standard provided. The colour system L*, a* and b* was used for analysis. L* represents lightness (L* = 0 for black, L* = 100 for white), a* indicates red/green (+a* intensity in red and –a* intensity in green) and b* represents yellow/blue (+b* intensity in yellow and –b* intensity in blue) [28]. Values of Chroma (colour intensity) and Hue angle (composed colour) were also obtained in the measurement. It is difficult to present standard colour values for a healthy/desirable muscle due to the great variability observed between studies; nevertheless, muscle colour in gilthead sea bream seems to be strongly related to its fat content [29], [30].

For texture analysis, a slab of ∼2 cm^2^ of skinless flesh, containing fast- and slow-twitch skeletal muscle, was dissected from the anterior-dorsal side of the fish to the dorsal spines and immediately kept on ice until analysis, 24 h later. Thickness of the muscle fillet was taken into consideration for normalisation of the texture data. Texture analysis was done using a TA.XT2i texturometer and a Mini Kramer/Ottawa cell blade in the Escola Superior d’Agricultura de Barcelona (ESAB) facilities, (Castelldefels, Spain). Total work, maximal strength and elasticity of flesh fragments from 4–8 fish per diet were analysed.

### RNA extraction and cDNA synthesis

Total RNA was extracted from ∼100 mg of fast muscle and between 40 to 500 mg of the other tissues from the tissue screening experiment, following the guanidinium thiocyanate-phenol-chloroform method [31] using TRIreagent (Applied Biosystems, Alcobendas, Spain). Total RNA was quantified using a NanoDrop2000 spectrophotometer (Thermo Scientific, Alcobendas, Spain), quality was verified as 260/280 and 260/230 ratios were over 1.8 in both cases and RNA integrity was analysed by 1% (m/v) agarose gel electrophoresis. To eliminate any residual genomic DNA, total RNA was treated with DNase I (Invitrogen, Alcobendas, Spain) following the manufacturer’s recommendations before cDNA synthesis. One µg of total RNA per sample was used to synthesise first-strand cDNA using the Transcriptor First Strand cDNA Synthesis Kit (Roche, Sant Cugat del Valles, Spain) following the manufacturer’s recommendations. cDNA samples were diluted 1∶5 in milliQ H_2_O for conventional polymerase chain reaction (RT-PCR) and diluted 1∶100 in milliQ H_2_O for real*-*time quantitative PCR (qPCR).

### Calpains cloning and sequencing

To obtain the complete sequences of *Sparus aurata* calpain1 (*sacapn1*), calpain2 (*sacapn2*), calpain3 (*sacapn3*), calpain small subunit1a (*sacapns1a*), primers for 5’ rapid amplification of cDNA ends (RACE)-PCR and specific primers were designed from gilthead sea bream ESTs (Expressed Sequence Tag) NCBI database (*sacapn1*: AM951595.1; *sacapn2*: FM155301.1, FG591123.1, FM152855.1 and FG265085.1; *sacapn3*: FG262721.1; *sacapns1a*: AM962179.1 and FM145762) and from 5’RACE-PCR amplicon products for *sacapn1* and *sacapn2* (Table S1). Also, *sacapn3* and calpain small subunit1b (*sacapns1b*) sequences were retrieved from the gilthead sea bream muscle transcriptome performed using 454 pyrosequencing (accession number: ERP000874) previously described by Garcia de la serrana *et* al., [32].

PCR products were separated by gel electrophoresis and purified using a PureLink Quick Gel Extraction Kit, ligated into T/A pCR4-TOPO vector and transformed into chemically competent TOP10 *Escherichia coli* cells by thermal shock (all from Invitrogen, Alcobendas, Spain). At least 1–3 clones of each PCR product were sequenced in both T3/T7 orientations using BigDye Terminator v3.1 Cycle Sequencing Kit (Applied Biosystems, Alcobendas, Spain) and analysed at the Serveis Cientificotècnics of the University of Barcelona (Barcelona, Spain). Sequenced products were joined *in silico* using DNAMAN (Lynnon Corporation, Quebec, Canada) to produce contigs with a single open reading frame (ORF). 5′RACE-PCR reactions were performed using a 5’RACE System for Rapid Amplification of cDNA Ends (Invitrogen, Alcobendas, Spain) following the manufacturer’s recommendations.

### Tissue screening

Qualitative RT-PCR was used to analyse calpains transcripts expression in different tissues. Elongation factor 1-alpha (*ef1α*) was used as a control gene. Reactions were performed in a final volume of 50 µL, containing 1 µL of first-strand cDNA (equivalent to 4 ng of reverse transcribed total RNA), 1.5U of Taq polymerase (Sigma, Tres Cantos, Spain) and 200 nM (final concentration) of sense and antisense primers (Table S2). Reactions proceeded in a C1000 Thermal Cycler (Bio-Rad, El Prat de Llobregat, Spain) with the following protocol: 1 cycle at 95°C for 5 min, 35 cycles at 95°C for 30 s, 53–61°C (primer dependent, see Table S2) for 30 s, 72°C for 0.5–1.5 min and 1 cycle at 72°C for 7 min. Each reaction product was separated by agarose gel electrophoresis and visualised using SYBR Safe DNA gel stain (Life Technologies, Alcobendas, Spain) in a LAS-3000 (Fujifilm, Madrid, Spain) to confirm that a single product was amplified, and then sequenced to confirm the specificity of each assay.

### Quantitative real-time PCR

The mRNA transcript levels of gilthead sea bream calpain genes (*sacapn1*, *sacapn2*, *sacapn3*, *sacapns1a* and *sacapns1b*), the β proteasome subunit N3 (*N3*) plus three reference genes (*ef1α*, beta-actin (*β-actin*) and ribosomal protein L27a (*rpl27a*)) were assessed using qPCR across the fasting/re-feeding and diet experiments. Each qPCR reaction contained 5 µL of first-strand cDNA (equivalent to 2.5 ng of reverse transcribed total RNA), 10 µL of iQ SYBR Green Supermix (Bio-Rad, El Prat de Llobregat, Spain) and 250 nM (final concentration) of sense and antisense primers (Table S3) in a final volume of 20 µL. Reactions were performed in triplicate using a MyiQ thermocycler (Bio-Rad, El Prat de Llobregat, Spain) with 1 cycle of 3 min at 95°C and 40 cycles of 10 s at 95°C and 30 s at 56–68 °C (primer dependent, see Table S3), followed by an amplicon dissociation analysis from 55 to 95°C at 0.5°C increase each 30 s, where a single peak was observed confirming the specifity of the reaction and the absence of primer-dimers formation. Also, prior to the analyses, a dilution curve with a pool of samples was run to confirm primer efficiency and to determine the appropriate cDNA dilution. SYBR Green fluorescence was recorded during the annealing-extending phase of cycling. Negative controls (NTC: No Template Control; RTC: no Reverse Transcriptase Control and PCR: water) were included and ran in duplicate. Raw data were normalized to *β-actin*, the most stable of the three reference genes analysed, by the delta-delta method [33].

### Statistical analyses

Statistical analyses of all parameters were performed in PASW Statistics 17.0 (IBM, Chicago, USA). Normality was analysed according to the Shapiro-Wilk test and homogeneity in variance according to Levene’s test. Therefore, statistical differences were assessed by one-way ANOVA, followed by Tukey’s test, or t-test. Non-parametric tests, Kruskal-Wallis and U de Mann-Whitney, were used when after data transformation normality was not found. A significance of p<0.05 was applied to all statistical tests performed. Data are presented as mean±standard error of the mean (SEM). Correlation analyses were carried out on the dietary experiment data in order to determine whether flesh texture was related to calpains expression, or whether any of the other variables also had an effect. Non-homoscedasticity was found; therefore the Spearman’s rank correlation coefficient (ρ) was performed. Correlation was considered significant at the bilateral levels of 0.05(*) or 0.01(**).

### Bioinformatic resources

Sequences used in the study other than those from gilthead sea bream were obtained from either NCBI (http://www.ncbi.nlm.nih.gov/) or ENSEMBL (http://www.ensembl.org) databases. Human tissue expression patterns of calpains were obtained from the GeneNote database ([34], http://genecards.weizmann.ac.il/genenote/). BLAST searches were performed against the NCBI non-redundant protein database (http://www.ncbi.nlm.nih.gov/blast). PSORTII [35] was used to predict nuclear localisation signals (NLSs) and Reinhardt’s method for Cytoplasmic/Nuclear discrimination ([36], http://psort.hgc.jp/form2.html). Compute pI/Mw tool (ExPASy, Switzerland, http://www.expasy.org/tools/pi_tool.html) was used to estimate the molecular weight (Mw) of the predicted proteins. In addition, polypeptide sequences rich in Proline (P), Glutamic acid (E), Serine (S) and Threonine (T) (PEST) that mark proteins as targets for rapid destruction were identified using the PEST finding program (http://mobyle.pasteur.fr/cgi-bin/portal.py#forms::epestfind).

A phylogenetic tree of 48 complete amino acid sequences of calpain large subunits (Calpains 1, 2, 3, 8, 9 and 11) and small subunits (Calpains 1a and 1b), from different vertebrates was performed. Calpain8, Calpain9 and Calpain11 were included, since these family members form a sister group to Calpain2, Calpain3 and Calpain1, respectively [23], [25], [37]. Calpain sequences were initially aligned using the Mafft v.6 (http://mafft.cbrc.jp/alignment/server/index.html) and G-INS-i (recommended for <200 sequences with global homology) strategy. Evolutionary analyses were conducted in MEGA5 [38]. The evolutionary history was inferred by using the Maximum Likelihood method based on the JTT matrix-based model [39]. The bootstrap consensus tree inferred from 1000 replicates was taken to represent the evolutionary history of the taxa analysed [40], and a discrete Gamma distribution was used to model evolutionary rate differences among invariant sites (G+I). The tree was drawn to scale, with branch lengths measured in the number of substitutions per site. The Atlantic salmon, *Salmo salar* cathepsin L (NM_001146546), a lysosomal cysteine protease, was used to root the phylogenetic tree.

## Results

### Calpains characterisation

The cDNA sequences of five distinct gilthead sea bream calpains were obtained using PCR, 5’RACE-PCR and 454 pyrosequencing and deposited in GenBank. BLAST searches were used to examine the identity of these new sequences.

The first complete coding region of 2118 base-pairs (bp) corresponding to a single ORF of 705 amino acids (aa) and a theoretical Mw of 79.9 kDa, returned highest BLAST scores to calpain1 sequences, showing 68% and 86% identity with human (*Homo sapiens*) and Atlantic salmon capn1, respectively; therefore, it was named *sacapn1* (accession number: KF444899) (Figure S1). A 2094 bp contig, coding a 697 aa protein with a Mw 78.2 kDa, showed 63% and 90% identity with mouse and Atlantic halibut capn2, respectively, and it was named *sacapn2* (accession number: KF444900) (Figure S2). Next, a 2316 bp contig, with a single ORF of 771 aa and 89.1 kDa Mw, showed 66% identity with mouse and 86% identity with halibut capn3 and was named *sacapn3* (accession number: ERP000874) (Figure S3).

Regarding the regulatory calpains, two 651 bp contigs, with single ORFs of 216 aa and theoretical Mw of 24.6 and 24.7 kDa, respectively, returned highest BLAST scores to calpain small subunit1 sequences. The first one showed 82% and 88% identity with zebrafish (NM_001017899.2) and Atlantic salmon (BT043754.1) capns1a and capns1, respectively and it was named *sacapns1a* (accession number: KF444901) (Figure S4). The second sequence showed 72% and 75% identity with Atlantic salmon (BT047225.1) and zebrafish (BC162479.1) capns1 and capns1b, respectively, indicating it was a sacapns1 paralog in gilthead sea bream; and thus it was named *sacapns1b* (accession number: ERP000874) (Figure S5).

**Figure 1:**
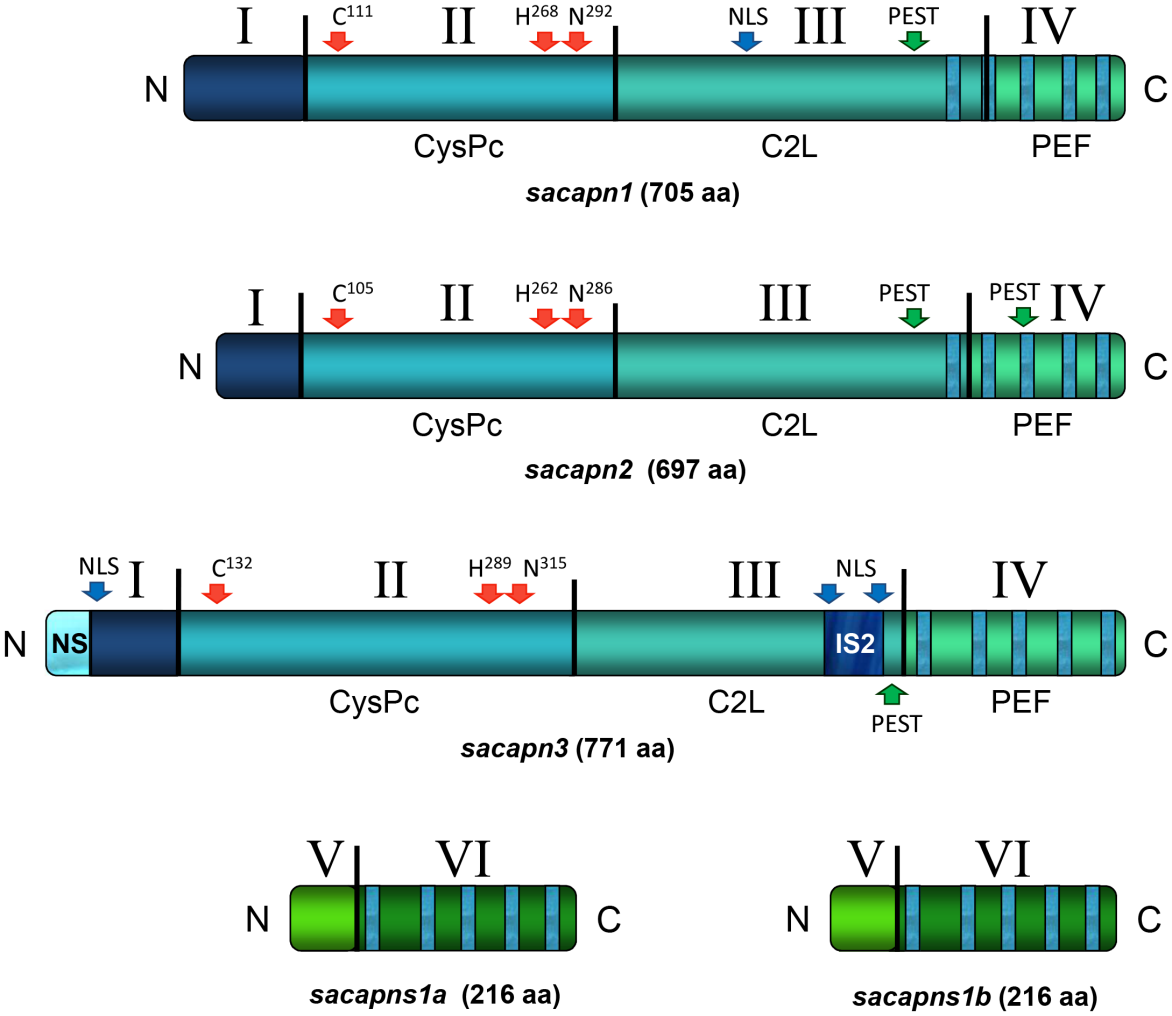
Schematic representation of the gilthead sea bream Calpain peptides’ structural architecture. Domains are identified with roman numbers. CysPc: proteolytic domain, C2L: C2-like domain, and PEF: penta-EF-hand domain. The conserved catalytic residues, nuclear localization signals (NLS) and PEST proteolytic signals are indicated with arrows.

The calpain domain architecture was identified in all five gilthead sea bream calpain peptides (Figure 1). Sacapn1 and Sacapn2 contained four domains (D): DI or the N-terminal anchor helix region, DII or the CysPc protease domain, DIII or the C2-like domain (C2L), and DIV or the penta-EF-hand domain (PEF) (Figures S1 and S2). Also, two additional regions were present in Sacapn3: the teleost N-terminal sequence (NS) and an insertion sequence (IS2) (Figure S3). The characteristic triad of catalytic residues, the potential PEST proteolytic signals that target proteins for rapid destruction and the nuclear localization signal (NLS) were also identified. Both paralogs of the regulatory calpain, Sacapns1a and Sacapns1b, contained two domains: DV and DVI or PEF domain (Figures S4 and S5). Finally, analysis of the calpain amino acid sequences using the PSORTII program predicted that all were cytoplasmic proteins.

### Phylogenetic analysis

**Figure 2:**
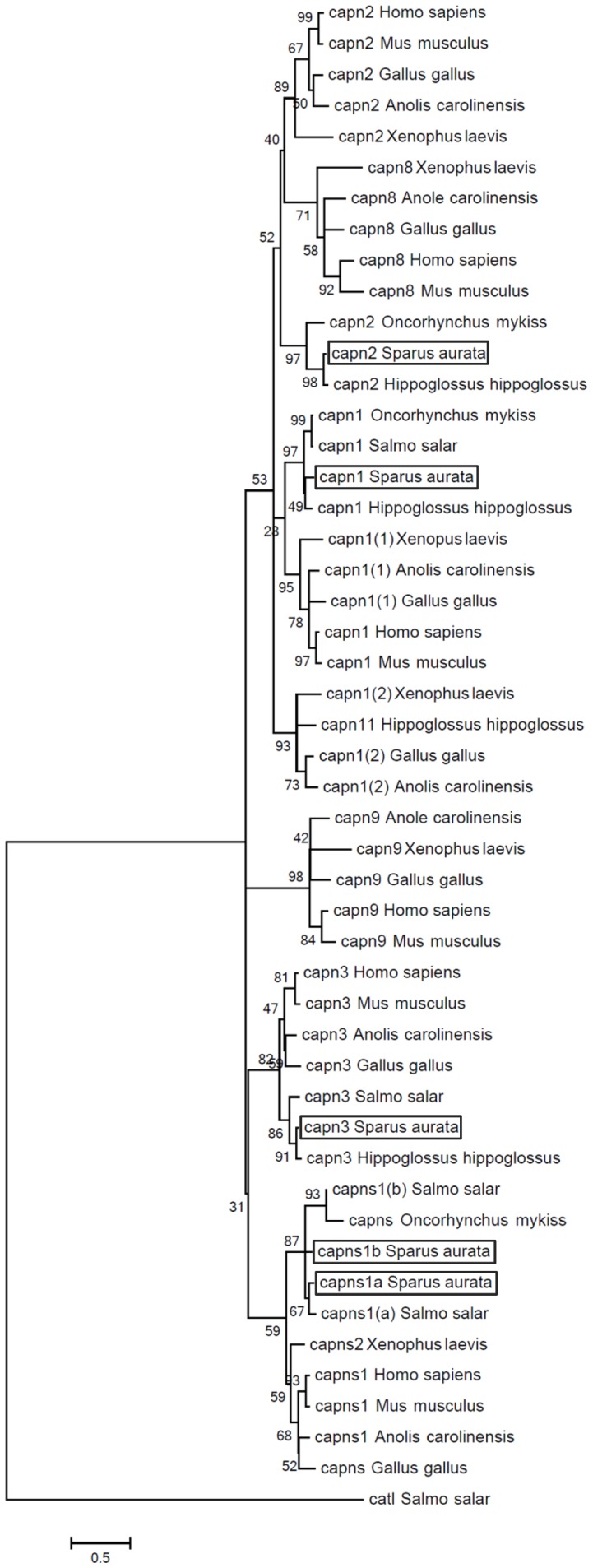
Molecular phylogenetic analysis of the gilthead sea bream Calpain amino acid sequences. A rooted phylogenetic tree of gilthead sea bream (*Sparus aurata*), Anole lizard (*Anolis carolinensis*), chicken (*Gallus gallus*), Atlantic halibut (*Hippoglossus hippoglossus*), human (*Homo sapiens*), mouse (*Mus musculus*), rainbow trout (*Oncorhynchus mykiss*), Atlantic salmon (*Salmo salar*) and African clawed frog (*Xenopus laevis*) Calpain1 (capn1), Calpain2 (capn2), Calpain3 (capn3), Calpain8 (capn8), Calpain9 (capn9), Calpain11 (capn11), and Calpain small subunits 1a and 1b (capns1a and capns1b) orthologs was performed. The Atlantic salmon Cathepsin L, a lysosomal cysteine protease, was used to root the phylogenetic tree. The tree was created by UPGMA method using ClustalW multiple alignment and bootstrapped 1000 times. The scale of the given branch length indicates 0,5 amino acid substitutions per site. Accession numbers were retrieved from public databases: *Anolis carolinensis* capn1(1) XM_003229570; capn1(2) XM_003215899; capn2 XM_003216038; capn3 XM_003214560; capn8 ENSACAT00000003160; capn9 ENSACAT00000002035 and capns1 XM_003228375; *Gallus gallus* capn1(1) NM_001044672; capn1(2) NM_205303; capn2 FJ232590; capn3 FJ232591; capn8 ENSGALT00000015288; capn9 ENSGALT00000018152 and capns AB007824; *Hippoglossus hippoglossus* capn1 GQ327965; capn2 GQ327966; capn3 GQ327967 and capn11 GQ327964; *Homo sapiens* capn1 BC075862; capn2 NM_001748; capn3 BC146649; capn8 NM_001143962; capn9 ENST00000271971 and capns1 ENST00000246533; *Mus musculus* capn1 AF021847; capn2 AF015038; AF127766; capn8 ENSMUST00000048941; capn9 ENSMUST00000093033 and capns1 ENSMUST00000001845; *Oncorhynchus mykiss* capn1 AY573919; capn2 NM_001124491 and capns NM_001124331; *Salmo salar* capn1 BT059271; capn3 NM_001165408; capns1(a) BT043754; capns1(b) BT047225 and cathepsin L1 (catl1) NM_001146546; *Sparus aurata* capn1 (KF444899); capn2 (KF444900); capn3 (ERP000874); capns1a (KF444901) and capns1b (ERP000874); *Xenopus laevis* capn1(1)NM_001087016; capn1(2)NM_001013613; capn2 NM_001090244; capn8 NM_001088543; capn9 NM_001092528 and capns2 BC078469.

A phylogenetic tree including 48 calpain sequences from different vertebrates, and an Atlantic salmon Cathepsin L sequence, was performed (Figure 2). The calpain cluster was divided into two main groups, one including the calpain large subunits (Capn1, Capn2, Capn8 and Capn11) and the second containing Capn3 and the calpain small regulatory subunits (Capns1a and Capns1b), whereas Capn9 formed a separated clade. Each putative gilthead sea bream calpain sequence was related to the corresponding calpain teleost ortholog, with both Capns paralogs forming two different sister clades.

### Tissue expression

**Figure 3:**
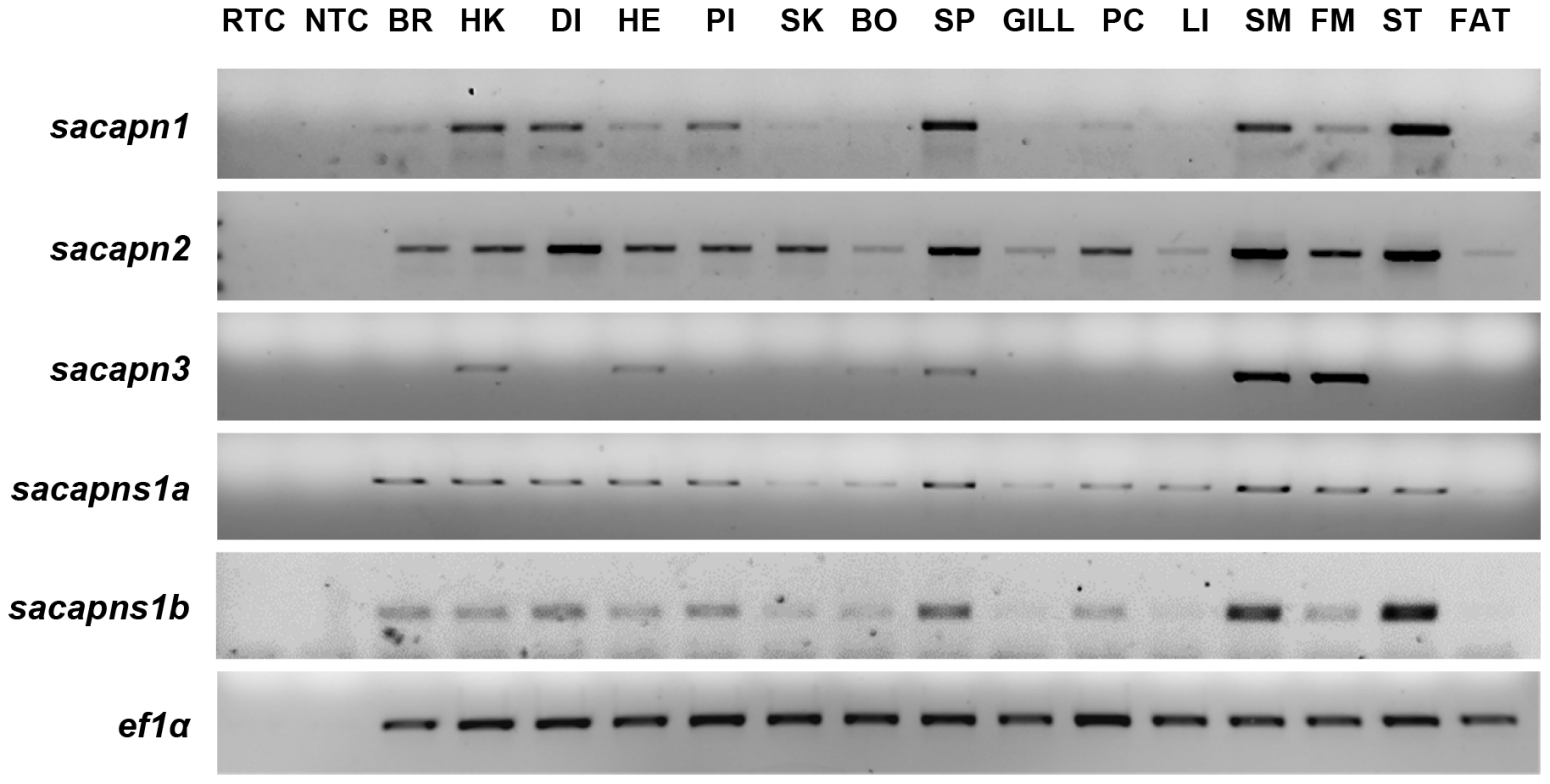
Tissue distribution of gilthead sea bream calpains. Qualitative transcript expression profiles of *sacapn1*, *sacapn2*, *sacapn3*, *sacapns1a*, *sacapns1b* and *ef1α*. RTC: No Reverse Transcriptase Control, NTC: No Template Control, BR: Brain, HK: Head kidney, DI: Distal intestine, HE: Heart, PI: Proximal intestine, SK: Skin, BO: Bone, SP: Spleen, GILL, PC: Pyloric caeca, LI: Liver, SM: Slow skeletal muscle, FM: Fast skeletal muscle, ST: Stomach, FAT: adipose tissue. A representative experiment from n = 3 independent fish analysed is shown.

Conventional RT-PCR was used to determine the mRNA expression of each gilthead sea bream calpain identified in 15 different tissues (Figure 3). Transcripts of *sacapn1*, *sacapn2*, *sacapns1a* and *sacapns1b*, were detected to a greater or lesser extent in each one of the 15 tissues examined. On the other hand, transcripts for *sacapn3* were detected preferentially in tissues containing striated muscle fibres, including fast and slow skeletal muscle and heart (Figure 3).

### Fasting/re-feeding experiment

Calpain gene expression was analysed in fast skeletal muscle of gilthead sea bream subjected to a fasting/re-feeding experiment. Fasting for 15 days caused a minor reduction in body mass and MFI, but decreased significantly HSI. During the same period, a significant increase was observed in body weight and MFI in the control group (Table 2). Again after 7 days of re-feeding the increase in body mass and MFI was not significant, although it was for HSI (Table 2).

**Table 2:** Biometrics of gilthead sea bream from the fasting and re-feeding experiment.

Condition^4^	Body weight (g)	Total length (cm)	HSI^1^	MFI^2^	CF^3^
D0C	50,79±2,13^a^	15,40±0,22	1,19±0,08^a^	0,35±0,05^a^	1,39±0,05^a^
D15C	57,28±4,26^ab^	16,00±0,39	1,25±0,09^a^	0,43±0,06^ab^	1,38±0,02^a^
D15F	46,32±1,70^a^	15,21±0,18	0,63±0,03^b^	0,32±0,04^a^	1,31±0,01^a^
D30C	61,77±2,18^b^	15,84±0,17	1,24±0,08^a^	0,60±0,10^b^	1,55±0,04^b^
D30F	46,91±1,98^a^	15,20±0,18	0,58±0,06^b^	0,29±0,03^a^	1,34±0,03^a^
D0F	46,91±1,98	15,20±0,18	0,58±0,06^a^	0,29±0,03	1,34±0,03
D7R	47,07±2,43	15,18±0,32	0,94±0,10^b^	0,30±0,04	1,34±0,03
D14R	47,09±1,52	15,44±0,25	0,89±0,06^b^	0,25±0,06	1,29±0,05

Body weight, total length, ^1^hepatosomatic index [HSI = (Liver weight/Body weight)*100], ^2^mesenteric fat index [MFI = (Adipose weight/Body weight)*100], and ^3^condition factor [CF = (Body weight/Total length^3^)*100]. Results are shown as mean ± SEM (n = 7–8). Different letters (^a,b^) indicate significant differences at p<0,05 with fasting and re-feeding periods analysed separately. ^4^Condition: D: day, C: control fed fish, F: fasted fish, R: re-fed fish.

As expected, plasmatic glucose was significantly reduced, while FFAs and TGs were significantly increased after 15 and 30 days of fasting (Table 3). In re-fed fish significantly elevated plasma glucose and reduced FFAs and TGs were observed (Table 3). In relation to muscle colour, no clear changes were observed during fasting for any of the parameters analysed; however, re-feeding significantly increased lightness (L*) and decreased a* and b* components towards green and blue intensities, respectively (Table S4).

**Table 3:** Plasma parameters of gilthead sea bream from the fasting and re-feeding experiment.

Condition^1^	Glucose (mg/dL)	FFAs (mEq/L)	TGs (mg/dL)
D0C	58,89±3,12^a^	0,35±0,04^a^	228,30±12,08^a^
D15C	56,65±4,18^ab^	0,25±0,03^a^	233,36±17,06^a^
D15F	43,89±1,39^c^	0,55±0,03^b^	441,28±67,95^b^
D30C	58,90±2,72^a^	0,23±0,03^a^	235,73±13,05^a^
D30F	46,52±1,54^bc^	0,55±0,03^b^	906,87±98,09^c^
D0F	46,52±1,54^a^	0,55±0,03^a^	906,87±98,09^a^
D7R	61,73±3,29^b^	0,39±0,04^b^	500,15±80,93^b^
D14R	64,51±2,93^b^	0,33±0,03^b^	262,26±41,54^b^

Results are shown as mean ± SEM (n = 7–8). Different letters (^a,b,c^) indicate significant differences at p<0,05 with fasting and re-feeding periods analysed separately. ^1^Condition: D: day, C: control fed fish, F: fasted fish, R: re-fed fish.

**Figure 4:**
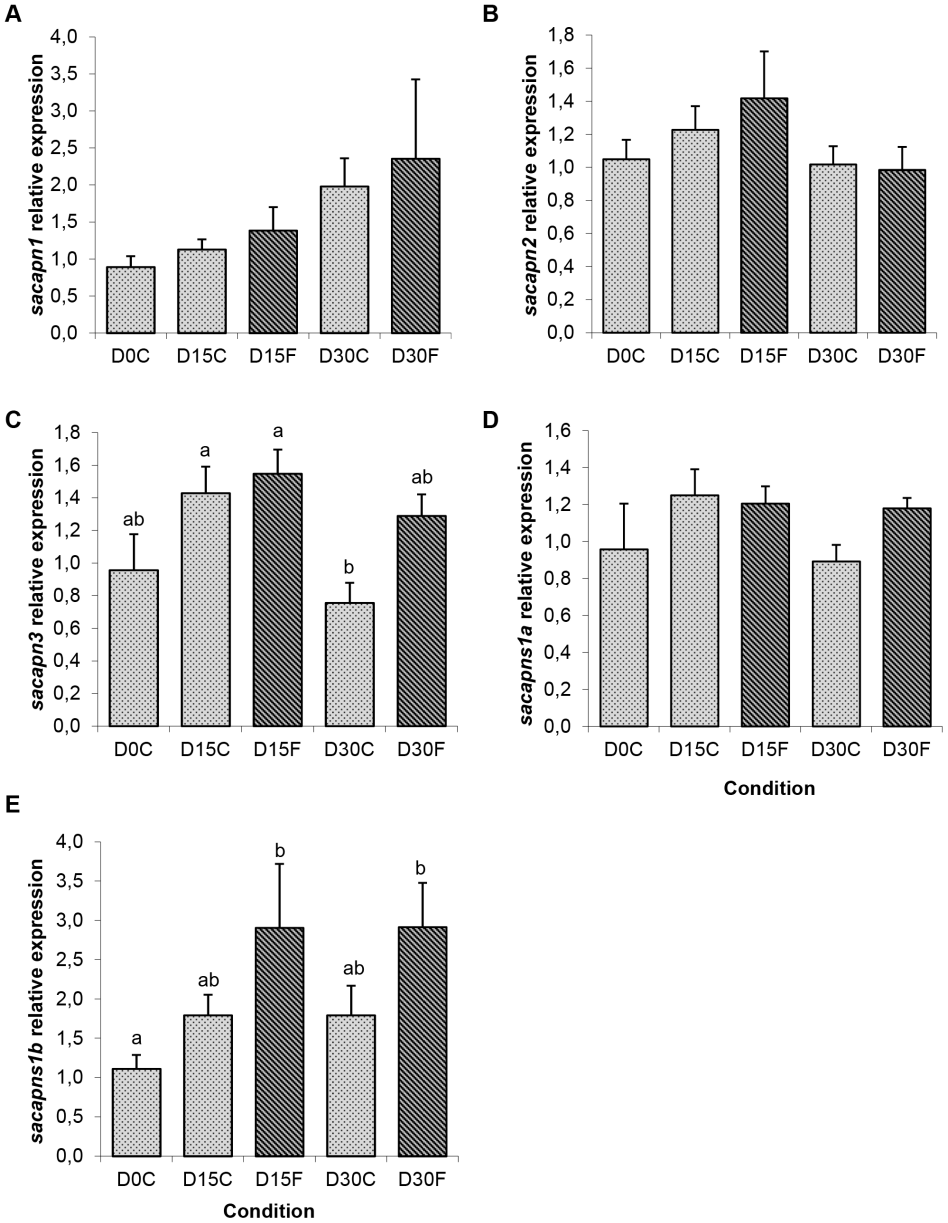
Calpains relative expression in gilthead sea bream from the fasting experiment. Quantitative expression relative to *β-actin* of (A) *sacapn1*, (B) *sacapn2*, (C) *sacapn3*, (D) *sacapns1a* and (E) *sacapns1b*. Results are shown as mean ± SEM (n = 5–8). Different letters indicate significant differences at p<0,05. C: control fed fish, F: fasted fish.

**Figure 5:**
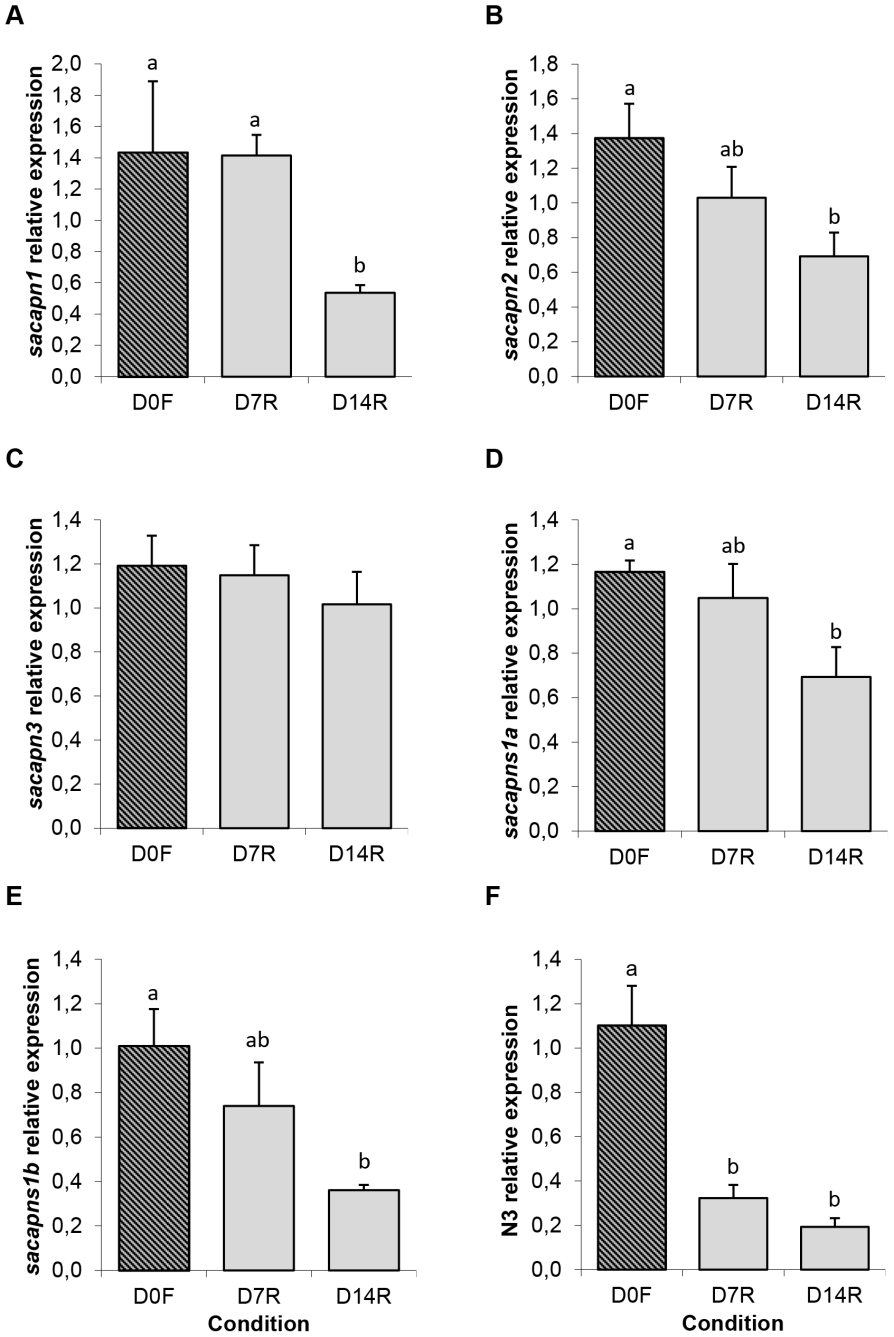
Calpains relative expression in gilthead sea bream from the re-feeding experiment. Quantitative expression relative to β-actin of (A) *sacapn1*, (B) *sacapn2*, (C) *sacapn3*, (D) *sacapns1a*, (E) *sacapns1b* and (F) *N3*. Results are shown as mean ± SEM (n = 5–8). Different letters indicate significant differences at p<0,05. F: fasted fish, R: re-fed fish.

Interestingly, fish fasted for 15 and 30 days presented a significant increase in *sacapns1b* expression, whereas the other calpains remained unchanged (Figure 4). Moreover, re-fed fish after 14 days had significantly decreased expression of *sacapn1*, *sacapn2*, *sacapns1a* and *sacapns1b*; and also a significant decrease was observed already at 7 days after re-feeding in the expression of the proteolysis marker of the proteasome, *N3* (Figure 5).

### Diet experiment

Then, we analysed whether the gilthead sea bream calpain genes identified were transcriptionally regulated according to the different percentages of dietary protein and CH (Table 1) on fast-twitch skeletal muscle. Finally, we evaluated if the expression of any of the calpains correlated with muscle texture. At the end of the experiment, no significant differences were found in SGR between the 46% protein groups (46/11 and 46/19). Nevertheless, differences were found respect fish fed the diets with 46% protein and those fed the other diets (Table 4). Also, significant differences were observed between fish fed the 42/35 and 40/39 diets, with the fish fed the 40/39 diet showing the smallest SGR. In addition, no significant differences in FCR between diets 46/11 and 42/35 were found; however, besides all groups had equal feed intake, differences were found between those two and the other groups (Table 4). Interestingly, the fish fed the diet 46/19 had the best FCR values, and the fish fed the 40/39 diet, the worst. Moreover, the fish fed with the diet 46/11 (with the lowest amount of CH), showed the highest final body weight and total length, which was significantly different with respect to fish fed with 42/35 and 40/39 diets, and showed significantly the lowest HSI value in comparison to the diets with higher amounts of CH (Table 5). The fish fed with the diet 40/39, showed the lowest MFI followed by those fed the diets 46/19 and 42/35, and showed the lowest CF, significantly different with respect to the fish fed the diet 46/19.

**Table 4:** Standard growth rate (SGR), feed intake and feed conversion rate (FCR) of gilthead sea bream fed the four experimental diets.

Diet	SGR^1^	Feed intake (g)	FCR^2^
46/11	1,14±0,01^a^	10961±593	1,61±0,07^a^
46/19	1,13±0,03^a^	9099±553	1,38±0,14^b^
42/35	1,05±0,02^b^	10157±356	1,73±0,06^a^
40/39	0,95±0,01^c^	10697±273	2,15±0,02^c^

1 Standard growth rate [SGR = (ln final weight (lnW_f_)-ln initial weight (lnW_i_))*100/time], [feed intake = feed offered-feed refused] and ^2^feed conversion rate [FCR = dry feed intake/wet weight gain]. Results are shown as mean ± SEM (n = 51). Different letters (^a,b,c^) indicate significant differences at p<0,05.

**Table 5:** Biometrics of gilthead sea bream fed the four experimental diets.

Diet	Body weight (g)	Total length (cm)	HSI^1^	MFI^2^	CF^3^
46/11	419,44±12,65^a^	24,01±0,23^a^	1,24±0,03^a^	1,06±0,12^ab^	3,03±0,07^ab^
46/19	396,78±15,69^ab^	23,16±0,19^b^	1,73±0,12^b^	1,32±0,20^a^	3,19±0,11^a^
42/35	363,10±8,64^b^	23,06±0,12^b^	2,12±0,12^b^	1,27±0,12^a^	2,96±0,07^ab^
40/39	271,70±7,11^c^	21,14±0,21^c^	1,93±0,18^b^	0,70±0,09^b^	2,83±0,04^b^

Body weight, total length, ^1^hepatosomatic index [HSI = (Liver weight/Body weight)*100], ^2^mesenteric fat index [MFI = (Adipose weight/Body weight)*100], and ^3^condition factor [CF = (Body weight/Total length^3^)*100]. Results are shown as mean ± SEM (n = 8–9). Different letters (^a,b,c^) indicate significant differences at p<0,05.

Plasma parameters, glucose and TGs were not significantly affected by diet composition. On the other hand, FFAs were significantly lower in the fish fed the 46/19 diet compared to the 46/11 and 40/39 groups (Table 6). Also, colour measurement of the dorsal muscle did not show any significant differences between groups (Table S5).

**Table 6:** Plasma parameters of gilthead sea bream fed the four experimental diets.

Diet	Glucose (mg/dL)	FFAs (mEq/L)	TGs (mg/dL)
46/11	74,41±6,30	0,28±0,02^a^	329,26±22,62
46/19	79,27±6,86	0,19±0,02^b^	290,80±30,10
42/35	74,46±3,53	0,23±0,01^ab^	380,23±54,07
40/39	91,50±12,45	0,28±0,02^a^	340,69±53,73

Results are shown as mean ± SEM (n = 8–9). Different letters (^a,b^) indicate significant differences at p<0,05.

**Figure 6:**
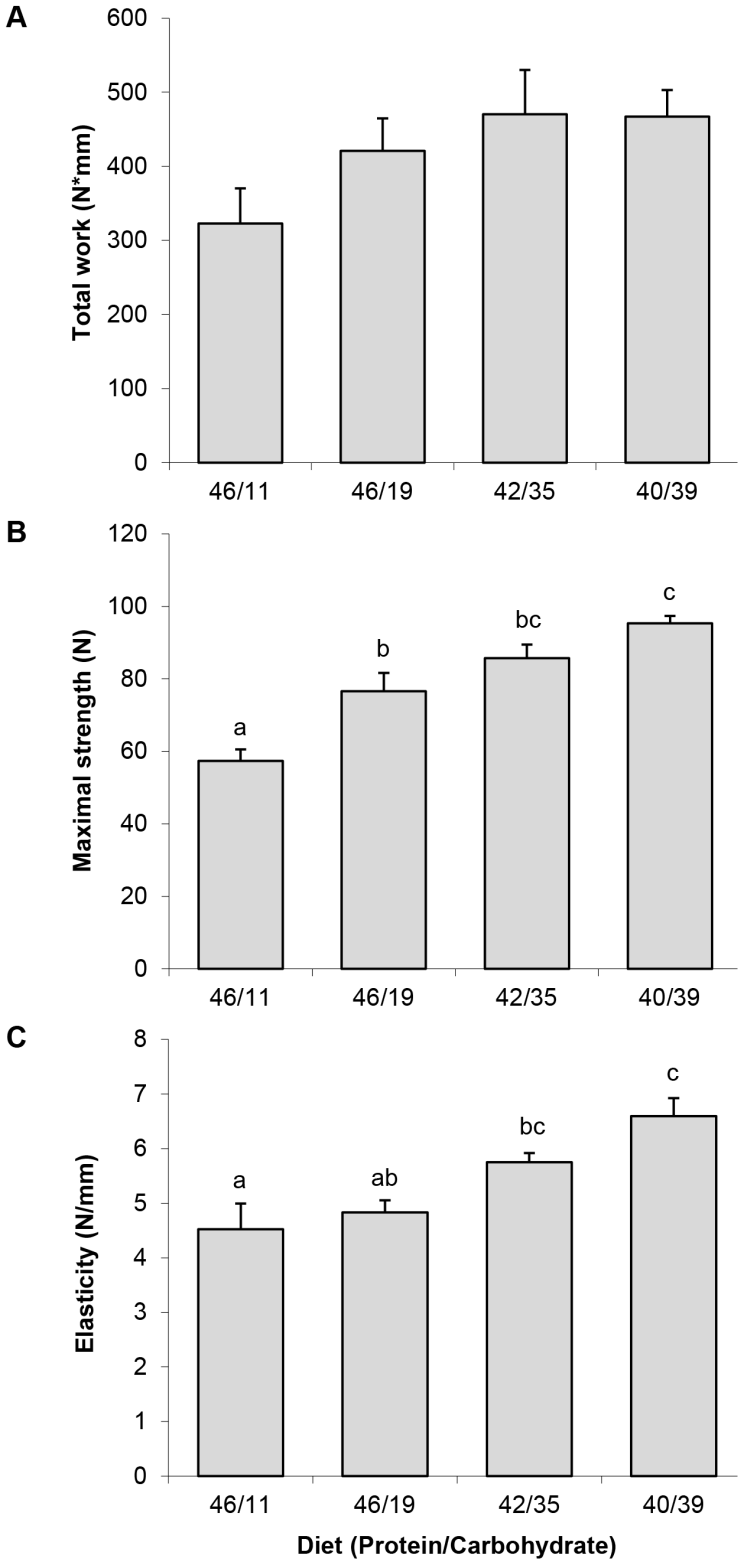
Fast skeletal muscle texture analysis. (A) total work, (B) maximal strength and (C) elasticity of gilthead sea bream fed the four experimental diets. Results are shown as mean ± SEM (n = 5–8). Different letters indicate significant differences at p<0,05.

**Figure 7:**
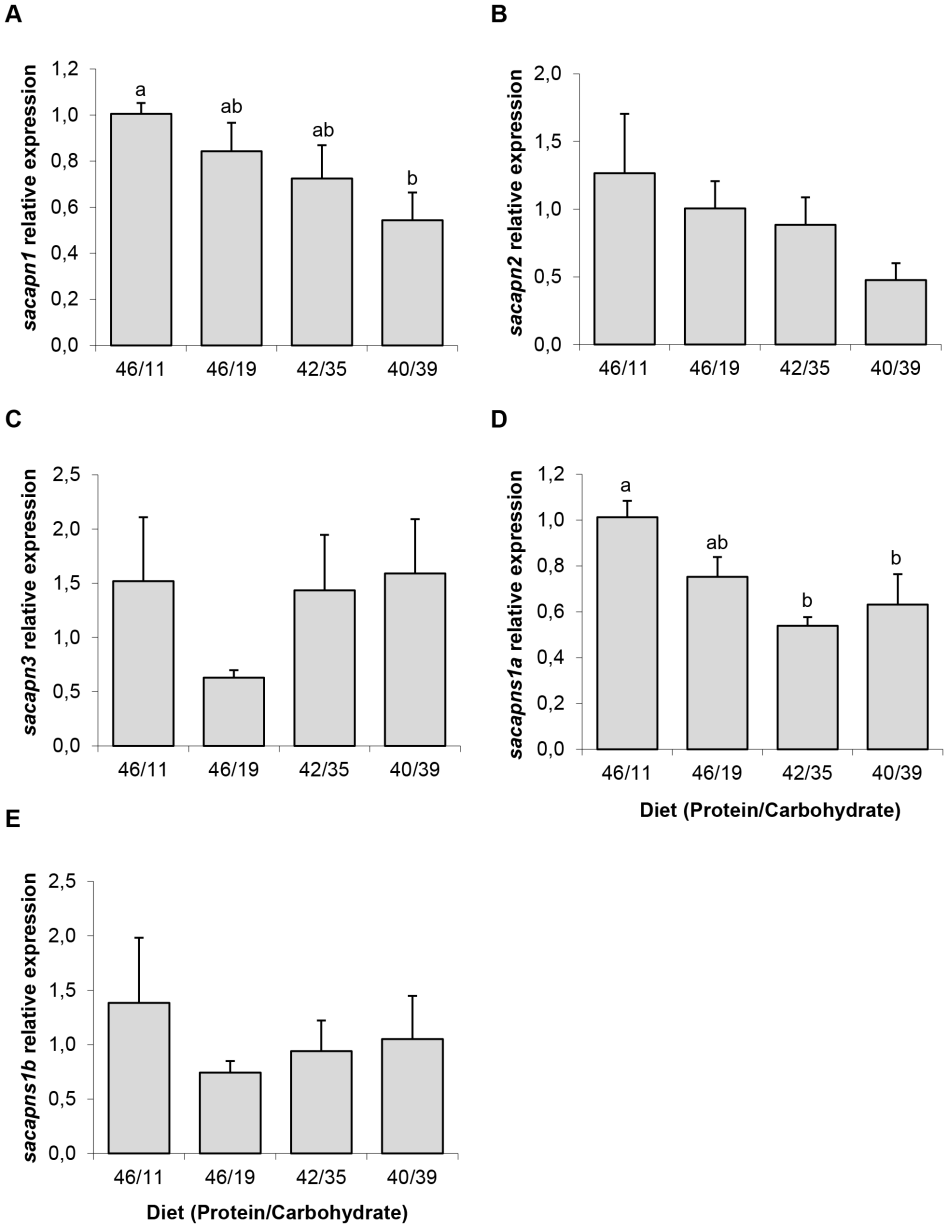
Calpains relative expression in gilthead sea bream fed the four experimental diets. Quantitative expression relative to *β-actin* of (A) *sacapn1*, (B) *sacapn2*, (C) *sacapn3*, (D) *sacapns1a* and (E) *sacapns1b*. Results are shown as mean ± SEM (n = 6–8). Different letters indicate significant differences at p<0,05.

The fish fed the 46/11 diet, showed significantly lower values in maximal strength and elasticity, while the diet 40/39 was associated with maximal values of both parameters and a firmer flesh. Total work showed a trend to increase with reduced dietary protein content, but the differences were not statistically significant (Figure 6). Fish fed the 46/11 diet had the highest relative expression of *sacapn1* and *sacapns1a*, while the diets 40/39 and 42/35 showed significantly lower values (Figure 7). The same decreasing trend was observed for *sacapn2* expression concomitantly with the increase of dietary CH, although no significant differences were found.

To better understand the possible relationship between calpains gene expression and flesh texture, correlations between these different parameters were performed. Significant Spearman ρ values of negative correlation were found between maximal strength and *sacapn1* as well as *sacapns1a* expression (ρ = –0.409* and ρ = –0.449*, respectively). Regarding calpain expression, significant positive correlations were observed between: *sacapn1* and *sacapn2* (ρ = 0.414*), *sacapn1* and *sacapns1a* (ρ = 0.814**), *sacapn2* and *sacapns1b* (ρ = 0.592**), *sacapn3* and *sacapns1a* (ρ = 0.455*) and *sacapns1a* and *sacapns1b* (ρ = 0.489*).

## Discussion

### Calpains characterisation

In the present study, several members of the calpain system have been characterised for the first time in gilthead sea bream (*Sparus aurata*) fast-twitch skeletal muscle. The typical calpain domain architecture was identified in all five gilthead sea bream calpains, in which the triad of catalytic residues common to all calpains was in each case highly conserved in comparison to vertebrates [19]. In agreement with Atlantic halibut [22], Sacapn3 lacks the IS1 domain present in tetrapods at the C-terminal region of DII [41], which altogether with NS and IS2 has to be autocatalytically removed for Calpain3 to become proteolitically active [42]. Regarding the regulatory subunits, in the N-terminal region of DV there is missing the Gly-rich and hydrophobic region characteristic of mammalian calpain small subunits that plays a role in membrane targeting, which was previously described absent in rainbow trout calpain small subunit [43] and in zebrafish calpain small 2-like [23], suggesting divergent function and activation mechanisms of the fish calpain system compared to mammals.

### Phylogenetic analysis

To further characterise the gilthead sea bream calpains, a phylogenetic tree was constructed. Importantly, each putative gilthead sea bream calpain sequence was related to the corresponding teleost ortholog as expected, as well as with its tetrapod ortholog with the exception of Calpain2. The teleost Calpain2 group formed a monophyletic clade external to the tetrapod Calpain2/8 sister group; thus, supporting the hypothesis that they are a close related group (i.e. Calpain2-like) and the common ancestor of vertebrate Calpains2/8 [23], [25]. These phylogenetic data together with conserved protein structure support a similar role for these proteinases in muscle tenderisation.

### Tissue expression

Next, qualitative RT-PCR was used to identify the distinct tissue expression of each gilthead sea bream calpain. Transcripts of *sacapn1*, *sacapn2*, *sacapns1a* and *sacapns1b*, were ubiquitously expressed as observed in rainbow trout [21], [43], Atlantic halibut [22], rabbits [44] or humans ([16], for instance. Furthermore as in mammals, where *calpain3* is principally expressed in skeletal muscle [41], regulating protein turnover and maintaining the sarcomere integrity [45], the *sacapn3* transcripts were mostly detected in fast- and slow-twitch skeletal muscle, but also in the heart. Moreover, *sacapn3* was also expressed to a lesser extent in other tissue types as previously reported in Atlantic halibut [25]; thus, suggesting a possible broader physiological role for Calpain3 in teleosts in comparison to mammals.

### Effects of nutritional status

In the wild, many fish species including gilthead sea bream are adapted to long-term food deprivation. In response to fasting, fish mobilize energy materials stored in their tissues, and after long periods, when more readily available energy sources have been exhausted, skeletal muscle proteins can be also mobilized resulting in muscle atrophy [46]. On the other hand, in anabolic situations, food intake stimulates the synthesis of new proteins, and to a lesser extent, also its degradation to induce protein turnover and growth.

Morphological and plasma parameters in fasted fish confirmed the catabolic state. *Sacapns1b* expression significantly increased after 15 days of fasting, while *sacapn1*, *sacapn2*, *sacapn3* and *sacapns1a* expression remained unaffected. In a previous study in rainbow trout, fasting for 35 days significantly stimulated the expression of *calpain1* and *calpain2*, but not that of the calpain small subunit [21]. In Atlantic halibut, *calpain1* but not *calpain2* was up-regulated after 60 days of fasting [22], and the contrary occurred in channel catfish, where 35 days of fasting increased *calpain2*, while decreased *calpain1* expression [24]. After re-feeding, significant increases were observed in HSI and plasma parameters returned to normal values. Changes in muscle colour indicated an increase in lightness during re-feeding, which may be attributed to an increase in lipid content as previously reported [29]. Furthermore, *sacapn3* was again unchanged but the relative expression of *sacapn1*, *sacapn2*, *sacapns1a* and *sacapns1b* was significantly reduced, suggesting a decrease in muscle proteolysis under these conditions, a result supported by the significant decrease observed during re-feeding of the subunit β of the proteasome *N3*, previously used in other studies as a proteolysis marker [47], [48]. These results were in agreement with the study in Atlantic halibut, where it was also observed that after 7 days of re-feeding, fish that were fasted for 60 days had *calpain1* transcript levels significantly decreased, but no differences were observed in *calpain2* expression, whereas a significant increase in *calpain3* was found [22]. In addition, in a recent study in gilthead sea bream fasted for 4 days, *calpain3* expression also increased 5–6 fold 24 h after re-feeding and was maintained until 6 days later [49].

Overall, these data suggests that the regulation of calpain expression with fasting and re-feeding is species-specific. In our study, *sacapns1b* expression was sensitive to fasting and re-feeding increasing and decreasing respectively, suggesting this calpain could be a potential marker to identify nutritional status in gilthead sea bream.

### Effects of diet composition

In their natural diet, gilthead sea bream feed mainly on molluscs and crustaceans, but the presence of algae is common in its intestinal contents. This indicates that gilthead sea bream can use vegetables, rich in CH and fibre, as an energy source. Previous studies on gilthead sea bream have shown that it is not advisable to exceed 20% of CH in their diet, due to a persistent postprandial hyperglycemia, that can finally decrease growth [50]. In the present study, isolipidic diets with different percentages of protein and CH were used to determine if it is possible to increase CH over the limit of 20% without affecting growth, but more interestingly, to see if calpain relative expression could be related to muscle texture, a parameter that can be modulated according to dietary treatment. Interestingly, although no differences were observed in feed intake or plasma glucose between groups, gilthead sea bream showed decreased growth parallel to the amount of protein in the diet. Fish fed diets 42/35 and 40/39 obtained significantly lower SGR and final body weight values compared to the fish fed the other diets (46/11 and 46/19). These results are in agreement with a previous study [50] and support the limited value of 20% CH dietary inclusion to achieve good growth rates in this species.

Texture analysis showed that in diets with 46% of protein, increasing CH levels up to 19% improved significantly flesh maximal strength; and an increase up to 35%, with a reduction of protein from 46 to 40%, elevated also significantly flesh elasticity. In previous studies in *Dentex dentex* it was also observed that a decrease in dietary protein content from 43 to 38% improved textural parameters as firmness and water holding; however, it was observed that within each dietary protein level, diets with high CH and low lipid content resulted in lower values of muscle firmness than diets with low CH [51]. Therefore, this is the first study in *Sparids* where an increase in dietary CH seems to improve textural parameters.

*Sacapn1* and *sacapns1a* were transcriptionally affected by the diet, while *sacapn2*, *sacapns1b* and *sacapn3* remained unaffected. Both, *sacapn1* and *sacapns1a* relative expression decreased with dietary CH increase and protein decrease, suggesting a reduction in muscle proteolysis and an increase in muscle texture in these fish. In agreement with this observation, *calpain1* mRNA levels were significantly lower in pigs fed a protein-free diet in comparison to control pigs [52]. In rainbow trout, it was observed that the level of *cathepsin D* expression in fast muscle increased by substitution of dietary fishmeal by a mix of plant protein sources, but *calpain2* was not modified [6]. Using different rainbow trout strains with distinct growth rates and fillet firmness and fed with two different energy diets, Salem *et* al., [26] reported that strain or diet did not affect the level of mRNAs expression for any of the calpain members analysed; however, significantly lower *calpastatin* expression was observed in the strain with softest fillet. These results suggest that the effects of diet on calpain expression show significant variation between fish species.

To further investigate the importance of the different gilthead sea bream calpains in flesh firmness, correlation analysis between texture parameters and calpains expression was performed. Interestingly, *sacapn1* and *sacapns1a* relative expression levels were significantly negatively correlated with maximal strength in our study. In mice, *calpain1* knockout animals had significantly reduced proteolysis in comparison to control mice [53]. Also, other studies in mammals have supported that calpain1, but not calpain2, is primarily responsible for meat tenderisation in beef and lamb [54], [55]; and in cattle, single nucleotide polymorphisms (SNPs) for the *calpain1* gene have been clearly associated with tenderness [56]. This has currently led to the use of markers within the *calpain1* as well as the *calpastatin* gene to identify the genetic potential of beef cattle to produce tender meat [57], a tool that is commercially available as a genetic test (GeneSTAR, Pfizer Genetics). The present results have revealed the potential use of calpains, *sacapn1* and *sacapns1a*, as candidate genes to monitor muscle growth and fillet firmness in gilthead sea bream.

In addition, *sacapn1* and *sacapn2* relative expression was significantly positively correlated, as both genes followed the same trend and decreased expression with an increased CH:protein ratio in the diet. A significant positive correlation between both *sacapns* paralogs was also found. Furthermore, the expression of small subunit paralogs, *sacapns1a* and *sacapns1b*, revealed also that each paralog was significantly correlated with each one of the catalytic calpains (*sacapns1a* with *sacapn1* and *sacapns1b* with *sacapn2*). Contrary to what it is observed in mammals, where the different catalytic calpains bind a common regulatory subunit to be fully active [19], the present data suggests that the genome duplication that occurred in the teleost lineage resulted in each fish catalytic calpain binding to a specific calpain regulatory subunit paralog. In order to confirm this hypothesis, further studies at the protein level will be required.

Finally, a significant positive correlation was found between the expression of paralog *sacapns1b* and *sacapn3*, suggesting a different regulation for calpain3 activity in fish, because in mammals, the recombinant PEF domain of Calpain3 is known to form a stable homodimer, but it is believed not to form a heterodimer with the calpain small subunit [58]. Moreover, the muscle-specific *calpain3* did not show differences in response to dietary treatment and did not correlate with muscle texture. In mammals, a strong correlation has been shown between *calpain3* mRNA levels and tenderness in cattle and sheep, whereas no correlation was reported in pigs [59], [60]. Also, it has been shown that Calpain3 can cleave calpastatin and the ubiquitous calpains, suggesting a role for calpain3 as an endogenous regulator of calpain expression and proteolytic activity [61]; thus indicating calpain3 deserves further attention in future studies in fish.

In summary, the present data has shown that several gilthead sea bream calpains are expressed in tissues with a distribution similar to that of calpains already described in other fish species as well as in mammals. We have also shown for the first time in teleosts the presence of two paralogs of the calpain small subunit (*sacapns1a* and *sacapns1b*) and the data has suggested that they are differently activated; *sacapns1b* with fasting and *sacapns1a* with changes in diet composition. Furthermore, the present results suggested that the expression of each paralog may be related to the expression of a corresponding catalytic subunit (*sacapns1a* with *sacapn1* and *sacapns1b* with *sacapn2*). Finally, we can conclude that the expression of some gilthead sea bream calpain genes, such as *sacapn1* and *sacapns1a*, may serve as potential genetic markers of flesh quality in this species.

## Supporting Information

Figure S1 **Complete ORF and deduced amino acid sequence of gilthead sea bream calpain1 (** ***sacapn1*** **).** The initiation and stop codons are shown in bold. The conserved catalytic residues are boxed and underlined. ↑ Indicates the boundaries of domains. The nuclear localization signal (NLS) is boxed in black. PEST proteolytic signal is boxed in grey. The penta-EF-hand (PEF) sequences are underlined.
(DOCX)

Figure S2 **Complete ORF and deduced amino acid sequence of gilthead sea bream calpain2 (** ***sacapn2*** **).** The initiation and stop codons are shown in bold. The conserved catalytic residues are boxed and underlined. ↑ Indicates the boundaries of domains. PEST proteolytic signals are boxed in grey. The penta-EF-hand (PEF) sequences are underlined.
(DOCX)

Figure S3 **Complete ORF and deduced amino acid sequence of gilthead sea bream calpain3 (** ***sacapn3*** **).** The initiation and stop codons are shown in bold. The teleost N-terminal sequence (NS) is shown in italics and underlined. Inserted sequence IS2 is shown in italics and boxed in pale grey. The conserved catalytic residues are boxed and underlined. ↑ Indicates the boundaries of domains. The nuclear localization signals (NLS) are boxed in black. PEST proteolytic signal is boxed in dark grey. The penta-EF-hand (PEF) sequences are underlined.
(DOCX)

Figure S4 **Complete ORF and deduced amino acid sequence of gilthead sea bream calpain small subunit1a (** ***sacapns1a*** **).** The initiation and stop codons are shown in bold. ↑ Indicates the boundaries of domains. The penta-EF-hand (PEF) sequences are underlined.
(DOCX)

Figure S5 **Complete ORF and deduced amino acid sequence of gilthead sea bream calpain small subunit1b (** ***sacapns1b*** **).** The initiation and stop codons are shown in bold. ↑ Indicates the boundaries of domains. The penta-EF-hand (PEF) sequences are underlined.
(DOCX)

Table S1 **Calpains primer sequences used for cloning by RT-PCR and 5' RACE-PCR.**
(DOCX)

Table S2 **Calpains primer sequences used for tissue screening by RT-PCR.**
(DOCX)

Table S3 **Calpains primer sequences used for qPCR.**
(DOCX)

Table S4 **Colour of gilthead sea bream muscle from the fasting and re-feeding experiment.** Colour measurements were performed on fast skeletal muscle from the antero-dorsal region. Colour is expressed using the L* (lightness), a* (red/green) and b* (yellow/blue) system. Results are shown as mean ± SEM (n = 7–8). Different letters indicate significant differences at p<0,05 with fasting and re-feeding periods analysed separately. C: control fed fish, F: fasted fish, R: re-fed fish.
(DOCX)

Table S5 **Colour of gilthead sea bream muscle fed the four experimental diets.** Colour measurements were performed on fast skeletal muscle from the antero-dorsal region. Colour is expressed using the L* (lightness), a* (red/green) and b* (yellow/blue) system. Results are shown as mean ± SEM (n = 7–9). No significant differences were observed at p<0,05.
(DOCX)
