# Supplementary material for: Characterisation and Expression of Calpain Family Members in Relation to Nutritional Status, Diet Composition and Flesh Texture in Gilthead Sea Bream (Sparus aurata)
Source: PLoS One. 2013 Sep 25;8(9):e75349. doi: 10.1371/journal.pone.0075349 (PMC3783371; doi:10.1371/journal.pone.0075349)
Supplement: Figure S1 — Complete ORF and deduced amino acid sequence of gilthead sea bream calpain1 ( sacapn1 ). The initiation and stop codons are shown in bold. The conserved catalytic residues are boxed and underlined. ↑ Indicates the boundaries of domains. The nuclear localization signal (NLS) is boxed in black. PEST proteolytic signal is boxed in grey. The penta-EF-hand (PEF) sequences are underlined. (DOCX) [file pone.0075349.s001.docx]

**Figure S1**

10 20 30 40 50 60

1 **ATG**GTGGAGCCCATTTGCGCCACCGGCATGGCCGCCAAGCTGAGGAGCCAGTGGGATCGC

1 **M** V E P I C A T G M A A K L R S Q W D R

70 80 90 100 110 120

61 GACGACGGCCTGGGGCAGAACCACAACGCCGTGAAGTTTCTGGGGCAGGACTTCGAGAGT

21 D D G L G Q N H N A V K F L G Q D F E S

130 140 150 160 170 180

121 CTTAAAGCCCAGTGCCTCCGGAGCGGGAAGCTGTTCGAGGATAGCTTGTTCCCCTGCGCC

41 L K A Q C L R S G K L F E D S L F P C A

190 200 210 220 230 240

181 GCGTCGTCACTGGGGTTCAAAGAGCTCGGCCCGAGATCCGCCAAGACCTACGGAGTCCGC

61 A S S L G F K E L G P R S A K T Y G V R

250 260 270 280 290 300

241 TGGATGAGGCCCACGGAGTTCTGCAAGCGCCCCGAGTTCATCGTGGACGGAGCTACTCGC

**↓**

81 W M R P T E F C K R P E F I V D G A T R

310 320 330 340 350 360

301 ACAGACATCTGTCAGGGAGCTCTAGGGGACTGCTGGCTGCTGGCGGCCATCGCCTCGCTC

101 T D I C Q G A L G D C W L L A A I A S L

370 380 390 400 410 420

361 ACCCTAAACGACAACCTGCTCCACAGAGTGGTTCCCCATGGACAGAGCTTCGGGCAGGGA

121 T L N D N L L H R V V P H G Q S F G Q G

430 440 450 460 470 480

421 TATGCTGGCATCTTTCACTTCCAGTTCTGGCAGTTTGGTGAGTGGGTGGAGGTGGTGATC

141 Y A G I F H F Q F W Q F G E W V E V V I

490 500 510 520 530 540

481 GACGACCGCCTGCCAGTGAAGGACGGGAAGCTGCTGTTCGTCCACTCGGCGGAGGGGACC

161 D D R L P V K D G K L L F V H S A E G T

550 560 570 580 590 600

541 GAGTTCTGGAGCGCCCTGCTGGAAAAGGCCTACGCCAAGTTGAACGGCTGCTACGAGGCC

181 E F W S A L L E K A Y A K L N G C Y E A

610 620 630 640 650 660

601 CTGTCAGGCGGCAGCACGTCGGAGGGCTTCGAGGACCTCACGGGCGGCGTGACGGAGATG

201 L S G G S T S E G F E D L T G G V T E M

670 680 690 700 710 720

661 TTTGAGCTGAGGAAAGCCCCGTCCGACCTCTTCAGCATCATCAGCCGGGCCATAGAGAGG

221 F E L R K A P S D L F S I I S R A I E R

730 740 750 760 770 780

721 GGGTCTCTGCTGGGCTGCTCCATCGACATCAGCAACACGTCGGACATGGAGGCCGTCACG

241 G S L L G C S I D I S N T S D M E A V T

790 800 810 820 830 840

781 TTCAAGAAGCTGGTGAAGGGACACGCCTACTCTGTGACCGGCGTGGAGGAGGTCGTGTAC

261 F K K L V K G H A Y S V T G V E E V V Y

850 860 870 880 890 900

841 AGAGGGAATCTGACCAAGCTGGTTCGCATCAGGAACCCCTGGGGTGAAGTGGAGTGGACC

281 R G N L T K L V R I R N P W G E V E W T

910 920 930 940 950 960

901 GGAGCCTGGAGCGACAACTCCAGAGAGTGGGACAGTGTGGATCGCTCCGTCAAGAGCCGG

301 G A W S D N S R E W D S V D R S V K S R

970 980 990 1000 1010 1020

961 CTCCAAAACCGCAGCGAGGGCGGAGAATTCTGGATGTCGTTCAGCGACTTCCTGCGCGAG

**↓**

321 L Q N R S E G G E F W M S F S D F L R E

1030 1040 1050 1060 1070 1080

1021 TTCACCCGCCTGGAGCTCTGCAACCTGACGGCCGACGCGCTGCAGAACAGCCAGCTGAAG

341 F T R L E L C N L T A D A L Q N S Q L K

1090 1100 1110 1120 1130 1140

1081 AAGTGGAGCTCCTCGCTGTATCAGGGGGAGTGGAGGAGAGGCAGCACGGCCGGAGGCTGC

361 K W S S S L Y Q G E W R R G S T A G G C

1150 1160 1170 1180 1190 1200

1141 AGGAACTACCCAGCAACCTTTTGGCTCAACCCTCAGTTCAAGCTCGTGCTGCAGCACCCG

381 R N Y P A T F W L N P Q F K L V L Q H P

1210 1220 1230 1240 1250 1260

1201 GACACTCCCGGCCAATCGGACTGCAGCTTCCTGGTCGGCCTCATGCAGAAGGACCGCAGG

401 D T P G Q S D C S F L V G L M Q K D R R

1270 1280 1290 1300 1310 1320

1261 AAGAAACGGCGGGAGGGCGAAGACATGGAGACCATCGGGTTCGCCCTCTATGAGGTTCCA

421 K K R R E G E D M E T I G F A L Y E V P

1330 1340 1350 1360 1370 1380

1321 AAGGAGTTTGTGGGCCGCTCAGGGGTCCACCTGAAGCGAGATTTTTTCCTCACCCACGCC

441 K E F V G R S G V H L K R D F F L T H A

1390 1400 1410 1420 1430 1440

1381 TCCAGCGCTCGCTCCGAGCTCTTCATCAACCTGAGGGAGGTTAGCTCGCGGTTGCGGCTG

461 S S A R S E L F I N L R E V S S R L R L

1450 1460 1470 1480 1490 1500

1441 CCGGCCGGGGAGTACGTCATCGTCCCCTCCACCTTCGAGCCGCACAAAGAGGCCGACTTC

481 P A G E Y V I V P S T F E P H K E A D F

1510 1520 1530 1540 1550 1560

1501 TGCCTCAGGGTCTTCTCGGAGAAGCCCGCCAACTCGGAGGAGCTGGACGATGATGTCGTA

501 C L R V F S E K P A N S E E L D D D V V

1570 1580 1590 1600 1610 1620

1561 GCGGATCTCCCAGCAGAGACCAAGCTGGACGAGAGCCAGATCGACTCCGGCTTCAAGAGT

521 A D L P A E T K L D E S Q I D S G F K S

1630 1640 1650 1660 1670 1680

1621 CTCTTCAGACAGCTGGCGGGGGCGGACATGGAGATCAGTATCACGGAGCTGCAAACCATA

541 L F R Q L A G A D M E I S I T E L Q T I

1690 1700 1710 1720 1730 1740

1681 TTGAACCGGATCATCAGCAAACACAAAGACCTGAAGACGGACGGCTTCACGAAAGAAGCC

561 L N R I I S K H K D L K T D G F T K E A

1750 1760 1770 1780 1790 1800

1741 TGTCGCAGCATGATAAACCTCATGGACACGGACGGCAGCGGGAAGCTCGGCCTGACGGAG

**↓**

581 C R S M I N L M D T D G S G K L G L T E

1810 1820 1830 1840 1850 1860

1801 TTCCACGTGCTCTGGGAGAAGATCAAACGATACCTGACCATATTCAGGGAGTTCGACCTG

601 F H V L W E K I K R Y L T I F R E F D L

1870 1880 1890 1900 1910 1920

1861 GACAAATCAGGCACCATGAGCTCCTACGAGATGAGGATGGCTCTCGATTCCGCAGGTTTC

621 D K S G T M S S Y E M R M A L D S A G F

1930 1940 1950 1960 1970 1980

1921 AAGCTGACCAACAACCTGTTCCAGCTGATCATCCTGCGCTACACGGAGGCCGACATGACC

641 K L T N N L F Q L I I L R Y T E A D M T

1990 2000 2010 2020 2030 2040

1981 GTCGACTTTGACAACTTTGTCACCTGCCTGGTCAGACTGGAGACCATGTACAAAACCTTT

661 V D F D N F V T C L V R L E T M Y K T F

2050 2060 2070 2080 2090 2100

2041 CAGACTCTGGACACAGATAAGGACAAAGTCATAGAGCTCAACTTCTTTCAGTGGATTACT

681 Q T L D T D K D K V I E L N F F Q W I T

2110

2101 CTGACCATGTTTGCC**TAG**

701 L T M F A *****
