# Supplementary material for: Characterisation and Expression of Calpain Family Members in Relation to Nutritional Status, Diet Composition and Flesh Texture in Gilthead Sea Bream (Sparus aurata)
Source: PLoS One. 2013 Sep 25;8(9):e75349. doi: 10.1371/journal.pone.0075349 (PMC3783371; doi:10.1371/journal.pone.0075349)
Supplement: Figure S2 — Complete ORF and deduced amino acid sequence of gilthead sea bream calpain2 ( sacapn2 ). The initiation and stop codons are shown in bold. The conserved catalytic residues are boxed and underlined. ↑ Indicates the boundaries of domains. PEST proteolytic signals are boxed in grey. The penta-EF-hand (PEF) sequences are underlined. (DOCX) [file pone.0075349.s002.docx]

**Figure S2**

10 20 30 40 50 60

1 **ATG**TCCGGCGTGGCCTCCACCCTGGCCAAGAAGCGGGCCCTGGCTGCGGGCTTCGGCACC

1 **M** S G V A S T L A K K R A L A A G F G T

70 80 90 100 110 120

61 AACGCCAATGCGACGCGGTACCTGAACCAGGACTTCAAGACCCTGCGGGCTCAGTGTAGT

21 N A N A T R Y L N Q D F K T L R A Q C S

130 140 150 160 170 180

121 TCCGCCGGGAAGCTGTTCTGCGACCCGACCTTCCCCGCCGCGCCCGAAGCGCTGGGCTTC

41 S A G K L F C D P T F P A A P E A L G F

190 200 210 220 230 240

181 AACGAGCTGGGCCGGAGCTCCTACAAGGTCCGCGGAGTCACCTGGAAGAGACCCACGGAA

**↓**

61 N E L G R S S Y K V R G V T W K R P T E

250 260 270 280 290 300

241 CTGGTCTCTAATCCTGAGTTCATCTTGGGCGGAGCCACTAGGACCGACATCTGCCAGGGT

81 L V S N P E F I L G G A T R T D I C Q G

310 320 330 340 350 360

301 GCTCTGGGTGACTGCTGGCTGTTGGCGGCCATCGCCTCGCTGACCCTTAACGAGTATGTG

101 A L G D C W L L A A I A S L T L N E Y V

370 380 390 400 410 420

361 ATGGCCAGAGTCGTTCCCACTGACCAGGGCTTCGGTGACGACTACGCCGGCATCTTCCAC

121 M A R V V P T D Q G F G D D Y A G I F H

430 440 450 460 470 480

421 TTCCAGTTCTGGCAGTTTGGTGAGTGGGTGGACGTGGTGATCGACGACCGCCTGCCGGTC

141 F Q F W Q F G E W V D V V I D D R L P V

490 500 510 520 530 540

481 AAAGATGGAGAGCTGATGTTCGTCCACTCGGCGGAGGGGAGGGAGTTCTGGAGCGCTCTG

161 K D G E L M F V H S A E G R E F W S A L

550 560 570 580 590 600

541 CTGGAGAAAGCCTACGCCAAAGTGAACGGCTGCTATGAAGCGCTGTCTGGCGGTTCCACC

181 L E K A Y A K V N G C Y E A L S G G S T

610 620 630 640 650 660

601 ACTGAAGGATTTGAAGATTTCACCGGTGGCATCGCTGAGAACTACGACCTCCAACGTCCC

201 T E G F E D F T G G I A E N Y D L Q R P

670 680 690 700 710 720

661 CCCGCCAACCTGTTCCAGATCATCAAGAAGGCCCTGGAGGCTGGAGCGCTGCTGGGCTGC

221 P A N L F Q I I K K A L E A G A L L G C

730 740 750 760 770 780

721 TCCATCGACATCACCAGCGCCGCAGACTCGGAGGCCGTCACCCGTCAGAAGCTGGTGAAA

241 S I D I T S A A D S E A V T R Q K L V K

790 800 810 820 830 840

781 GGCCACGCCTACTCACTGACGGGAGCTGTGGAGGTGAACTTCCGTGGCCGGAATGAGCGG

261 G H A Y S L T G A V E V N F R G R N E R

850 860 870 880 890 900

841 CTGGTGAGGATGAGGAACCCGTGGGGTCAGGTGGAGTGGACCGGAGCGTGGAGCGACGGA

281 L V R M R N P W G Q V E W T G A W S D G

910 920 930 940 950 960

901 TCGTCTGAGTGGAGCCAGGTGCAGGGAGACTGTCCACATGCCAACGCAGAGGACGGAGAG

**↓**

301 S S E W S Q V Q G D C P H A N A E D G E

970 980 990 1000 1010 1020

961 TTCTGGATGTCCTTCAGCGACTTCTGCCGTCACTATAATCGTGTTGAGTTGTGCACTCTG

321 F W M S F S D F C R H Y N R V E L C T L

1030 1040 1050 1060 1070 1080

1021 ACCCCCGACACCATCGAAGATGACTCTGTCAAACACTGGAGCGTCAGCAAGTTCGATGGC

341 T P D T I E D D S V K H W S V S K F D G

1090 1100 1110 1120 1130 1140

1081 TCCTGGAGGAGAGGATCCACCGCTGGAGGCTGCAGGAACCACCCTTACACGTTCTGGATG

361 S W R R G S T A G G C R N H P Y T F W M

1150 1160 1170 1180 1190 1200

1141 AATCCTCAGTTTGTGATCGAGCTGGATGAGGAGGATGATGACCCCGATGATGGCGAAGTG

381 N P Q F V I E L D E E D D D P D D G E V

1210 1220 1230 1240 1250 1260

1201 GGCTGCAGCTTTGTGGTCGGTCTGATCCAGAAGAACCGCAGAAAGCTCCGGAAACAAGGA

401 G C S F V V G L I Q K N R R K L R K Q G

1270 1280 1290 1300 1310 1320

1261 GAGGACATGCACACCGTTGGGTTTGCCATCTATGAGGTTCCAAAGGAGTATCAAGGCCAG

421 E D M H T V G F A I Y E V P K E Y Q G Q

1330 1340 1350 1360 1370 1380

1321 AGGGAGGTGCATCTGGACAAGAACTACTTCCTGACCCACGCTCAGACGGCAAAGTCCGAA

441 R E V H L D K N Y F L T H A Q T A K S E

1390 1400 1410 1420 1430 1440

1381 ACCTTCATCAACCTGCGTGAGGTCTGCTCTCGCTTCAAGCTGCCCCCAGGAGAGTACCTG

461 T F I N L R E V C S R F K L P P G E Y L

1450 1460 1470 1480 1490 1500

1441 ATCGTCCCGTCCACCTTCGAACCGCATCTCAATGGAGACTTCTGCATCCGTGTGTTCTCT

481 I V P S T F E P H L N G D F C I R V F S

1510 1520 1530 1540 1550 1560

1501 GAGAAGCAGACTGAGACCCAGCCCTGTGACGACCCGGTCCAGGCTGAACTAGATGATGAG

501 E K Q T E T Q P C D D P V Q A E L D D E

1570 1580 1590 1600 1610 1620

1561 ACGGTGTCTGATGAGGACGTGGACGCAGGGTTCAGAGGACTCTTCTCGAAACTCGCTGGA

521 T V S D E D V D A G F R G L F S K L A G

1630 1640 1650 1660 1670 1680

1621 GACGACATGGAGATCTCAGCGGTGGAGCTCAGGACCATCATGAACAAGATCGTCTCCAAA

**↓**

541 D D M E I S A V E L R T I M N K I V S K

1690 1700 1710 1720 1730 1740

1681 CGAACTGACATCAAAACTGACGGCTTCAGCCTGGAGACCTGCAGGGTCATGGTCAACCTG

561 R T D I K T D G F S L E T C R V M V N L

1750 1760 1770 1780 1790 1800

1741 ATGGATGACAGCGGGAACGGGAAGCTCGGCCTTGGAGAGTTCGCCACCTTGTGGAAGAAG

581 M D D S G N G K L G L G E F A T L W K K

1810 1820 1830 1840 1850 1860

1801 GTGCAGAGATACCTGTCCATCTATAAGAAGAACGACTCGGACAACTCGGGGACGATGAGC

601 V Q R Y L S I Y K K N D S D N S G T M S

1870 1880 1890 1900 1910 1920

1861 ACGCCGGAGATGAGAGTCGCCTTTAAAGACGCAGGTTTCAGCCTCAACAACACCATCTAC

621 T P E M R V A F K D A G F S L N N T I Y

1930 1940 1950 1960 1970 1980

1921 CAGCTGCTGGTGGCTCGATACTCCGACCCAGACATGACCATCGACTTCGACAACTTCGTG

641 Q L L V A R Y S D P D M T I D F D N F V

1990 2000 2010 2020 2030 2040

1981 GGCTGTCTGATGAGGCTGGAGATGATGTTCAGGATCTTCAAGAAGCTCGACGCTCAGGAC

661 G C L M R L E M M F R I F K K L D A Q D

2050 2060 2070 2080 2090

2041 AGCGGCTCCATCGAGCTCGACTTCAACCAGTGGTTAAACTTCGCCATGATC**TGA**

681 S G S I E L D F N Q W L N F A M I *****
