# Supplementary material for: Characterisation and Expression of Calpain Family Members in Relation to Nutritional Status, Diet Composition and Flesh Texture in Gilthead Sea Bream (Sparus aurata)
Source: PLoS One. 2013 Sep 25;8(9):e75349. doi: 10.1371/journal.pone.0075349 (PMC3783371; doi:10.1371/journal.pone.0075349)
Supplement: Figure S3 — Complete ORF and deduced amino acid sequence of gilthead sea bream calpain3 ( sacapn3 ). The initiation and stop codons are shown in bold. The teleost N-terminal sequence (NS) is shown in italics and underlined. Inserted sequence IS2 is shown in italics and boxed in pale grey. The conserved catalytic residues are boxed and underlined. ↑ Indicates the boundaries of domains. The nuclear localization signals (NLS) are boxed in black. PEST proteolytic signal is boxed in dark grey. The penta-EF-hand (PEF) sequences are underlined. (DOCX) [file pone.0075349.s003.docx]

**Figure S3**

10 20 30 40 50 60

1 **ATG**GGGGACGAGACATATAAAGGGAAAGTTCCTCTCGTTGAGGACACAAAGGTGAAAGTG

1 ***M*** *G D E T Y K G K V P L V E D T K V K V*

70 80 90 100 110 120

61 CTTTATGAGACTCAAGCATCAGCTGGGCCTGATGACAAAGCTGAATACCCTCCTGCTGGG

21 *L Y E T Q A S A G P D D K A E Y P P A G*

130 140 150 160 170 180

121 ACTAACTCCATCTACTCTGCGATTCTGAGCAGAAACGAGGCCGTCAAGGATGCCAAGCGC

41 *T N* S I Y S A I L S R N E A V K D A K R

190 200 210 220 230 240

181 CTTAAGACTTTTTTGGAGCTGCGAGACAAATACGTGAAGAAGAAGGTGGTGTTTGAAGAC

61 L K T F L E L R D K Y V K K K V V F E D

250 260 270 280 290 300

241 CCTCTGTTCCCCGCAAACGACTCCTCGCTCTTCTACAGTCACAAGTCTGCCATGAAGATC

81 P L F P A N D S S L F Y S H K S A M K I

310 320 330 340 350 360

301 GAGTGGAAGCGTCCCTCGGAAATTTGTGAAAACCCCCAGTTCATCATCGATGGAGCCAAT

**↓**

101 E W K R P S E I C E N P Q F I I D G A N

370 380 390 400 410 420

361 CGGACAGACATCTGTCAGGGAGAATTGGGTGACTGCTGGTTGCTGGCTGCCATCGCCTGT

121 R T D I C Q G E L G D C W L L A A I A C

430 440 450 460 470 480

421 CTGACAGTCAATGAGAAGCTGCTGTACAGAGTGATTCCCCCCGATCAGAGCTTCACTGAG

141 L T V N E K L L Y R V I P P D Q S F T E

490 500 510 520 530 540

481 AACTACGCTGGCATCTTCCATTTCCAGTTCTGGCGTTATGGCGAATGGATCGATGTGGTT

161 N Y A G I F H F Q F W R Y G E W I D V V

550 560 570 580 590 600

541 GTGGACGACCGCATCCCCACCTGCAACAACAAGCTGGTTTTCACCAAATCTTTCAGGAAT

181 V D D R I P T C N N K L V F T K S F R N

610 620 630 640 650 660

601 AACGAGTTCTGGAGCGCCCTTTTGGAAAAAGCTTACGCAAAGTTGCACGGGTCTTATGAG

201 N E F W S A L L E K A Y A K L H G S Y E

670 680 690 700 710 720

661 GCACTGAAAGGGGGCAACACCTTGGAAGCCATGGAGGATTTCACGGGTGGTGTTACGGAG

221 A L K G G N T L E A M E D F T G G V T E

730 740 750 760 770 780

721 TTCTTCGAGCTGTCTGAGGCGCCCAAAGACCTCTACAGCATCATGAGGAAGGCGCTGCAG

241 F F E L S E A P K D L Y S I M R K A L Q

790 800 810 820 830 840

781 AGAGGCTCGCTGATGGGCTGCTCCATAGATGTTTTTTCAGCCAGTGAACTGGAGTCTCGG

261 R G S L M G C S I D V F S A S E L E S R

850 860 870 880 890 900

841 ACTGATCTGGGGCTGGTGAGGGGTCATGCCTACTCCATCATCGGCCTGGAGGAGTGTGAC

281 T D L G L V R G H A Y S I I G L E E C D

910 920 930 940 950 960

901 GAAGTTGCAAAGAACACCAAAGTTCGCCTGATTCGCCTGCGCAATCCCTGGGGTTTCGTG

301 E V A K N T K V R L I R L R N P W G F V

970 980 990 1000 1010 1020

961 CTGTGGAAAGGACCATGGAGTGTAAATTCAAAGGAATGGTCGACCATTTCCACTGCAGAC

321 L W K G P W S V N S K E W S T I S T A D

1030 1040 1050 1060 1070 1080

1021 AGGGAAAACCTAAAGAAACAGACGATAGAAACGAGTGAGTTCTGGATGTCTTTTGATGAT

**↓**

341 R E N L K K Q T I E T S E F W M S F D D

1090 1100 1110 1120 1130 1140

1081 TTTAAGAGGAACTTCACCAAGCTGGAGATGTGTAACCTGACCCCTGACACACTGCAGTGT

361 F K R N F T K L E M C N L T P D T L Q C

1150 1160 1170 1180 1190 1200

1141 GATGAGAGACACAGCTGGACGGTGTCCGTCAATGAGGGTCGTTGGGTGAGGGGCAGCTCT

381 D E R H S W T V S V N E G R W V R G S S

1210 1220 1230 1240 1250 1260

1201 GCTGGAGGCTGCAGGAACTTCCCAGAAACGTTCTGGACGAACCCTCAGTATCGGCTGAAG

401 A G G C R N F P E T F W T N P Q Y R L K

1270 1280 1290 1300 1310 1320

1261 CTGTACGAAGAGGATGACGACCCAGAGGACGGGAACATGGCCTGCACTCTCGTTGTGGCT

421 L Y E E D D D P E D G N M A C T L V V A

1330 1340 1350 1360 1370 1380

1321 CTGATGCAGAAAGGTCGAAGGATGCAGCGTCATCAAGGAGCCAGATTCCTCACCATTGGA

441 L M Q K G R R M Q R H Q G A R F L T I G

1390 1400 1410 1420 1430 1440

1381 TTTTCCATCTACCAGGTCCCAAAGGAGATGTGTGGACAGAATCAGCATCTGCAGAAGGAC

461 F S I Y Q V P K E M C G Q N Q H L Q K D

1450 1460 1470 1480 1490 1500

1441 TTTTTCCTGTACACAGCCTCCAAGGCTAAATGCAAGACCTACATTAACCTGCGGGAGGTC

481 F F L Y T A S K A K C K T Y I N L R E V

1510 1520 1530 1540 1550 1560

1501 ACGGAGCGGTTCCGTCTGCCCCCGGGGGAGTATGTCATCATCCCCACGACCTTTCAACCT

501 T E R F R L P P G E Y V I I P T T F Q P

1570 1580 1590 1600 1610 1620

1561 CATCAAGAGGGAGAGTTCATTCTCAGGGTCTTCTCTGAGAAGCAGAGCACGTCTGAGGAA

521 H Q E G E F I L R V F S E K Q S T S E E

1630 1640 1650 1660 1670 1680

1621 GTGGAGAACACGATCGGCTCTGACCAAACACAGCAAGACAAGAAAAAGAAAGAAAAGCCT

541 V E N T I G S D *Q T Q Q D K K K K E K P*

1690 1700 1710 1720 1730 1740

1681 ATTGTATTTGTGTCAGACAGAGCACGAGCCAACAAAGAAATCGAGCATGACGGCATTCTG

561 *I V F V S D R A R A N K E I E H D G I L*

1750 1760 1770 1780 1790 1800

1741 GGAGAAAAGAAGAAGAAACCAAAGCGAAAATTACTTGAACCTGAGGAGGAGACTGAAGAG

581 *G E K K K K P K R K L* L E P E E E T E E

1810 1820 1830 1840 1850 1860

1801 GAAAAACAGTTCAGAGCCATTTACGAACAGATTGCTGGTGAAGACATGCAGATCTGTGCC

**↓**

601 E K Q F R A I Y E Q I A G E D M Q I C A

1870 1880 1890 1900 1910 1920

1861 AACGAACTTATGAAGGTCATGAAGAATGTCCTCGCCAAACATAGTGAAATAAAGGCAGAG

621 N E L M K V M K N V L A K H S E I K A E

1930 1940 1950 1960 1970 1980

1921 GGTTTCAGCCTTGAGACGTGTCGGAGCATGATTGCCCTGATGGATACTGATGGAACAGGA

641 G F S L E T C R S M I A L M D T D G T G

1990 2000 2010 2020 2030 2040

1981 AAGCTGAACCTGCAGGAGTTCAAACACTTGTGGAGAAAGATCAAAGCGTGGCAGCTGATC

661 K L N L Q E F K H L W R K I K A W Q L I

2050 2060 2070 2080 2090 2100

2041 TTCAAACGTTACGATAAGGACAAAACCTGCTCCATCAGTAGTTTTGAGATGAGAAACGCA

681 F K R Y D K D K T C S I S S F E M R N A

2110 2120 2130 2140 2150 2160

2101 GTTAATGATGCAGGGTTTCACCTCAACAACCAGTTATATGACATCATAGCCATGCGCTAC

701 V N D A G F H L N N Q L Y D I I A M R Y

2170 2180 2190 2200 2210 2220

2161 GCAGATGAACACCTCAACATCAACTTTGACAGTTACATCTGCTGTTTTGTGAGGCTAGAG

721 A D E H L N I N F D S Y I C C F V R L E

2230 2240 2250 2260 2270 2280

2221 GGCATGTTTAGGGCTTTCAATGCTTTTGACAAAGACGGTGATGGAATCATCAAACTGAAT

741 G M F R A F N A F D K D G D G I I K L N

2290 2300 2310

2281 GTCCTGGAGTGGCTCCAGCTGACTATGTATTCT**TAA**

761 V L E W L Q L T M Y S *****
