# Supplementary material for: Characterisation and Expression of Calpain Family Members in Relation to Nutritional Status, Diet Composition and Flesh Texture in Gilthead Sea Bream (Sparus aurata)
Source: PLoS One. 2013 Sep 25;8(9):e75349. doi: 10.1371/journal.pone.0075349 (PMC3783371; doi:10.1371/journal.pone.0075349)
Supplement: Figure S4 — Complete ORF and deduced amino acid sequence of gilthead sea bream calpain small subunit1a ( sacapns1a ). The initiation and stop codons are shown in bold. ↑ Indicates the boundaries of domains. The penta-EF-hand (PEF) sequences are underlined. (DOCX) [file pone.0075349.s004.docx]

**Figure S4**

10 20 30 40 50 60

1 **ATG**TTCTTTGCCAAAAAGTTTATCGGTGGCATCATTGATGTTGTCAGCAACATCGACCCA

1 **M** F F A K K F I G G I I D V V S N I D P

70 80 90 100 110 120

61 GCCCAGTTTGTCCCTTCTGAACCTCCTCCACCACGTAGACCCGCTGTATATGCAGAGCAG

21 A Q F V P S E P P P P R R P A V Y A E Q

130 140 150 160 170 180

121 CATGAGAGCGATGAGGAGAAACAATTTCGCAGAGTCTTCCAGCAACTCGCTGGGGATGAC

**↓**

41 H E S D E E K Q F R R V F Q Q L A G D D

190 200 210 220 230 240

181 ATGGAAGTGAGCCCATCAGAGCTGATGAACATACTGAACAGAATCATTGGAAAACATGGT

61 M E V S P S E L M N I L N R I I G K H G

250 260 270 280 290 300

241 GACCTGAAGACGGATGGTTTCAGCATTGAGTCTTGCAGGAGCATGGTCGCAGTCATGGAT

81 D L K T D G F S I E S C R S M V A V M D

310 320 330 340 350 360

301 AGTGACAGCACTGGAAAACTCGGCTTTCACGAATTCAAACACCTCTGGAACAATATAAAG

101 S D S T G K L G F H E F K H L W N N I K

370 380 390 400 410 420

361 AAGTGGCAGGGGGTGTATAAAGCCCATGACAGAGATGGCTCTGGTGTCATTGGTGCAGAT

121 K W Q G V Y K A H D R D G S G V I G A D

430 440 450 460 470 480

421 GAGTTGCCAGAAGCTTTCAGAGCTGCGGGCTTCCCCCTCAATGACCAGCTGTTCCAGATG

141 E L P E A F R A A G F P L N D Q L F Q M

490 500 510 520 530 540

481 ATAATTCGCAGATACAGCGATGAAAATGGGAACATGGATTTTGACAACTACATTGGCTGC

161 I I R R Y S D E N G N M D F D N Y I G C

550 560 570 580 590 600

541 CTTGTGAGGCTAGATGCCATGTGCCGTTCCTTCAAAACCCTGGATAAGGATAACAATGGG

181 L V R L D A M C R S F K T L D K D N N G

610 620 630 640 650

601 ACTATCAAAGTCAATGTTCAGGAGTGGCTTCAGTTGACCATGTACTCT**TGA**

201 T I K V N V Q E W L Q L T M Y S *****
