# Supplementary material for: Characterisation and Expression of Calpain Family Members in Relation to Nutritional Status, Diet Composition and Flesh Texture in Gilthead Sea Bream (Sparus aurata)
Source: PLoS One. 2013 Sep 25;8(9):e75349. doi: 10.1371/journal.pone.0075349 (PMC3783371; doi:10.1371/journal.pone.0075349)
Supplement: Figure S5 — Complete ORF and deduced amino acid sequence of gilthead sea bream calpain small subunit1b ( sacapns1b ). The initiation and stop codons are shown in bold. ↑ Indicates the boundaries of domains. The penta-EF-hand (PEF) sequences are underlined. (DOCX) [file pone.0075349.s005.docx]

**Figure S5**

10 20 30 40 50 60

1 **ATG**TTTATGGCGAAGGCGTTCATCAAAGGCCTCATCAATGTCGTGAGCGACATCGACCCG

1 **M** F M A K A F I K G L I N V V S D I D P

70 80 90 100 110 120

61 TCACAGTTTCGTCCTTCAGACCCTCCTCCACCTCGCAGACCTCTGAACTTCGCTGAGACT

21 S Q F R P S D P P P P R R P L N F A E T

130 140 150 160 170 180

121 CATGAGAGCGACGAGGAGCAAAAGTTTCGGAGGGTTTTCAAGCAGCTGGCCGGAGATGAT

**↓**

41 H E S D E E Q K F R R V F K Q L A G D D

190 200 210 220 230 240

181 ATGGAGGTGAGTCCTAAAGAGCTGATGGACATCCTCAACAAAATCGTTTCCAAACATGGA

61 M E V S P K E L M D I L N K I V S K H G

250 260 270 280 290 300

241 GGTCTGAAGACTGACGGCTTCAGCATCGAGTCCTGCAGGAGCATGGTGGCCGTCATGGAC

81 G L K T D G F S I E S C R S M V A V M D

310 320 330 340 350 360

301 AGCGACAGCACAGGGAAACTGGGCTTCCACGAGTTCAAATACCTCTGGAACAACATCAAG

101 S D S T G K L G F H E F K Y L W N N I K

370 380 390 400 410 420

361 AGATGGCAGGGTATTTATATGTCCCATGATGCCGATGGTTCAGGTGTGATCTGCGATCAA

121 R W Q G I Y M S H D A D G S G V I C D Q

430 440 450 460 470 480

421 GAGCTGCCGAAAGCCTTCAAGGCTGCAGGCTTCCCTCTGAACGACCAGCTCTTCAAGCTG

141 E L P K A F K A A G F P L N D Q L F K L

490 500 510 520 530 540

481 ATTATCCGTCGCTATAGCGATGAGCACGGCAACATGGACTTTGACAACTTCGTCGGCTGC

161 I I R R Y S D E H G N M D F D N F V G C

550 560 570 580 590 600

541 CTGGTGCGACTGGACGCCATGTGCAGAGCCTTTAAGACCCTGGACAAGGACAACAGTGGC

181 L V R L D A M C R A F K T L D K D N S G

610 620 630 640 650

601 ACCATAGACTTGGACATCAAGGAGTGGCTTCAGCTGACGATGTATTCA**TGA**

201 T I D L D I K E W L Q L T M Y S *****
