# Supplementary material for: Characterisation and Expression of Calpain Family Members in Relation to Nutritional Status, Diet Composition and Flesh Texture in Gilthead Sea Bream (Sparus aurata)
Source: PLoS One. 2013 Sep 25;8(9):e75349. doi: 10.1371/journal.pone.0075349 (PMC3783371; doi:10.1371/journal.pone.0075349)
Supplement: Table S1 — Calpains primer sequences used for cloning by RT-PCR and 5' RACE-PCR. (DOCX) [file pone.0075349.s006.docx]

**Table S1**

| **Gene** | **Assay** | **Sense strand primer (5'-3')** | **Antisense strand primer (5'-3')** | **Anneal temp (ºC)** | **Product size (bp)** |
| --- | --- | --- | --- | --- | --- |
| *sacapn1* | 5' RACE GSP1a |  | GTCCTTATCTGTGTCC |  |  |
|  | 5' RACE nested GSP2a | 5'RACE Abridged Anchor Primer | GTCTGACCAGGCAGGTGACAAAGTT | 55 |  |
|  | 5′ RACE nested GSPa | Abridged Universal Amplification Primer | GGTGCCCGATTTGTCCAGGT | 55 | 1006 |
|  | 5' RACE GSP1b |  | CTTTGGAACCTCATAG |  |  |
|  | 5' RACE nested GSP2b | 5'RACE Abridged Anchor Primer | CCTCCACTCCCCCTGATACA | 55 |  |
|  | 5′ RACE nested GSPb | Abridged Universal Amplification Primer | ATCCACACTGTCCCACTCTCTG | 55 | 425 |
|  | 5' RACE GSP1c |  | CATCTCTGGAGTTGTC |  |  |
|  | 5' RACE nested GSP2c | 5'RACE Abridged Anchor Primer | TGTTGCTGATGTCGATGGAG | 55 |  |
|  | 5′ RACE nested GSPc | Abridged Universal Amplification Primer | CTCGTAGCAGCCGTTCAACT | 55 | 467 |
|  | 5' RACE GSP1d |  | ATGTCTGTGCGAGTAG |  |  |
|  | 5' RACE nested GSP2d | 5'RACE Abridged Anchor Primer | CTTTGAACCCCAGTGACGAC | 55 |  |
|  | 5′ RACE nested GSPd | Abridged Universal Amplification Primer | CGCAGGGGAACAAGCTATC | 55 | 719 |
| *sacapn2* | RT-PCR | GCTCTGGGTGACTGCTGGCTG | GACATCCAGAACTCTCCGTCCTC | 64 | 671 |
|  | RT-PCR | GAGGACGGAGAGTTCTGGATGTC | CCACTGGTTGAAGTCGAGCTC | 61 | 1121 |
|  | 5' RACE GSP1 |  | ACTGGAAGTGGAAGAT |  |  |
|  | 5' RACE nested GSP2 | 5'RACE Abridged Anchor Primer | CTGGTCAGTGGGAACGACTC | 55 |  |
|  | 5′ RACE nested GSP | Abridged Universal Amplification Primer | ACAGCCAGCAGTCACCCAGA | 55 | 395 |
| *sacapn3* | RT-PCR | AGGGTTTCAGCCTTGAGACG | CTGGAGCCACTCCAGGACATT | 56 | 379 |
| *sacapns1a* | RT-PCR | TCCTCAACCTCAACAAAGTGC | GGAAGTGGTAGAGATGGTTGAGA | 56 | 783 |
