# Supplementary material for: Characterisation and Expression of Calpain Family Members in Relation to Nutritional Status, Diet Composition and Flesh Texture in Gilthead Sea Bream (Sparus aurata)
Source: PLoS One. 2013 Sep 25;8(9):e75349. doi: 10.1371/journal.pone.0075349 (PMC3783371; doi:10.1371/journal.pone.0075349)
Supplement: Table S2 — Calpains primer sequences used for tissue screening by RT-PCR. (DOCX) [file pone.0075349.s007.docx]

**Table S2**

| **Gene** | **Assay** | **Sense strand primer (5'-3')** | **Antisense strand primer (5'-3')** | **Anneal temp (ºC)** | **Product size (bp)** |
| --- | --- | --- | --- | --- | --- |
| *sacapn1* | RT-PCR | GAAGGACCGCAGGAAGAAAC | GTGCTACCGCTGGATTGACT | 58 | 1111 |
| *sacapn2* | RT-PCR | GAGGACGGAGAGTTCTGGATGTC | CCACTGGTTGAAGTCGAGCTC | 61 | 1122 |
| *sacapn3* | RT-PCR | TCCTCGCTCTTCTACAGTCAC | CGTAACACCACCCGTGAAAT | 58 | 455 |
| *sacapns1a* | RT-PCR | TTCCTCAACCTCAACAAAGTGC | CAAGGCAGCCAATGTAGTT | 56 | 590 |
| *sacapns1b* | RT-PCR | ATGTTTATGGCGAAGGCG | AGAGGTATTTGAACTCGTGGAAG | 53 | 346 |
| *ef1α* | RT-PCR | CTTCAACGCTCAGGTCATCAT | GCACAGCGAAACGACCAAGGGGA | 60 | 264 |
