# Supplementary material for: Characterisation and Expression of Calpain Family Members in Relation to Nutritional Status, Diet Composition and Flesh Texture in Gilthead Sea Bream (Sparus aurata)
Source: PLoS One. 2013 Sep 25;8(9):e75349. doi: 10.1371/journal.pone.0075349 (PMC3783371; doi:10.1371/journal.pone.0075349)
Supplement: Table S3 — Calpains primer sequences used for qPCR. (DOCX) [file pone.0075349.s008.docx]

**Table S3**

| **Gene** | **Assay** | **Sense strand primer (5'-3')** | **Antisense strand primer (5'-3')** | **Anneal temp (ºC)** | **Product size (bp)** |
| --- | --- | --- | --- | --- | --- |
| *sacapn1* | qPCR | CCTACGAGATGAGGATGGCT | AGTTGTCAAAGTCGGCGGT | 56 | 114 |
| *sacapn2* | qPCR | ACCCACGCTCAGACGGCAAA | CGTTCCCGCTGTCATCCATCA | 61 | 405 |
| *sacapn3* | qPCR | AGAGGGTTTCAGCCTTGAGA | CGCTTTGATCTTTCTCCACA | 56 | 113 |
| *sacapns1a* | qPCR | CGCAGATACAGCGATGAAAA | GTTTTGAAGGAACGGCACAT | 56 | 92 |
| *sacapns1b* | qPCR | ATGGACAGCGACAGCACA | AGAGGTATTTGAACTCGTGGAAG | 56 | 51 |
| *N3* | qPCR | AGACACACACTGAACCCGA | TTCCTGAAGCGAACCAGA | 54 | 119 |
| *ef1α* | qPCR | CTTCAACGCTCAGGTCATCAT | GCACAGCGAAACGACCAAGGGGA | 60 | 263 |
| *β-actin* | qPCR | TCCTGCGGAATCCATGAGA | GACGTCGCACTTCATGATGCT | 60 | 50 |
| *rpl27a* | qPCR | AAGAGGAACACAACTCACTGCCCCA | GCTTGCCTTTGCCCAGAACTTTGTAG | 68 | 159 |
