# Supplementary material for: Characterisation and Expression of Calpain Family Members in Relation to Nutritional Status, Diet Composition and Flesh Texture in Gilthead Sea Bream (Sparus aurata)
Source: PLoS One. 2013 Sep 25;8(9):e75349. doi: 10.1371/journal.pone.0075349 (PMC3783371; doi:10.1371/journal.pone.0075349)
Supplement: Table S4 — Colour of gilthead sea bream muscle from the fasting and re-feeding experiment. Colour measurements were performed on fast skeletal muscle from the antero-dorsal region. Colour is expressed using the L* (lightness), a* (red/green) and b* (yellow/blue) system. Results are shown as mean ± SEM (n = 7–8). Different letters indicate significant differences at p<0,05 with fasting and re-feeding periods analysed separately. C: control fed fish, F: fasted fish, R: re-fed fish. (DOCX) [file pone.0075349.s009.docx]

**Table S4**

| **Condition** | **L*** | **a*** | **b*** | **Chroma** | **Hue** |
| --- | --- | --- | --- | --- | --- |
| D0C | 44,77 ± 0,72^a^ | -1,36 ± 0,10 | -2,84 ± 0,30^a^ | 3,16 ± 0,30^a^ | 243,47 ± 1,90^a^ |
| D15C | 51,07 ± 0,62^c^ | -1,40 ± 0,07 | -2,47 ± 0,23^ab^ | 2,85 ± 0,22^ab^ | 239,67 ± 1,87^a^ |
| D15F | 49,82 ± 0,38^c^ | -1,46 ± 0,04 | -2,72 ± 0,31^ab^ | 3,11 ± 0,27^ab^ | 240,10 ± 2,97^ab^ |
| D30C | 48,69 ± 0,65^bc^ | -1,66 ± 0,08 | -1,14 ± 0,28^c^ | 2,11 ± 0,16^b^ | 211,57 ± 6,81^c^ |
| D30F | 46,80 ± 1,10^ab^ | -1,44 ± 0,09 | -1,63 ± 0,34^bc^ | 2,33 ± 0,26^ab^ | 231,50 ± 2,99^b^ |
| D0F | 46,80 ± 1,10^a^ | -1,44 ± 0,09^a^ | -1,63 ± 0,34^a^ | 2,33 ± 0,26^a^ | 231,50 ± 2,99 |
| D7R | 49,45 ± 1,23^ab^ | -1,72 ± 0,07^ab^ | -2,55 ± 0,14^ab^ | 3,10 ± 0,13^ab^ | 235,06 ± 1,51 |
| D14R | 50,75 ± 0,80^b^ | -1,77 ± 0,08^b^ | -2,90 ± 0,35^b^ | 3,42 ± 0,32^b^ | 237,04 ± 2,70 |
