# Supplementary material for: Characterisation and Expression of Calpain Family Members in Relation to Nutritional Status, Diet Composition and Flesh Texture in Gilthead Sea Bream (Sparus aurata)
Source: PLoS One. 2013 Sep 25;8(9):e75349. doi: 10.1371/journal.pone.0075349 (PMC3783371; doi:10.1371/journal.pone.0075349)
Supplement: Table S5 — Colour of gilthead sea bream muscle fed the four experimental diets. Colour measurements were performed on fast skeletal muscle from the antero-dorsal region. Colour is expressed using the L* (lightness), a* (red/green) and b* (yellow/blue) system. Results are shown as mean ± SEM (n = 7–9). No significant differences were observed at p<0,05. (DOCX) [file pone.0075349.s010.docx]

**Table S5**

| **Diet** | **L*** | **a*** | **b*** | **Chroma** | **Hue** |
| --- | --- | --- | --- | --- | --- |
| 46/11 | 45,82 ± 1,99 | 2,05 ± 0,27 | -3,35 ± 0,45 | 4,09 ± 0,33 | 298,78 ± 4,45 |
| 46/19 | 51,96 ± 2,12 | 2,65 ± 0,32 | -2,80 ± 0,56 | 4,17 ± 0,32 | 315,98 ± 7,77 |
| 42/35 | 47,85 ± 2,77 | 3,07 ± 0,48 | -2,38 ± 0,44 | 4,11 ± 0,31 | 320,63 ± 8,47 |
| 40/39 | 50,34 ± 1,89 | 2,48 ± 0,20 | -2,82 ± 0,22 | 3,84 ± 0,29 | 317,65 ± 5,31 |
